# Mathematical Details on a Cancer Resistance Model

**DOI:** 10.3389/fbioe.2020.00501

**Published:** 2020-06-17

**Authors:** James M. Greene, Cynthia Sanchez-Tapia, Eduardo D. Sontag

**Affiliations:** ^1^Department of Mathematics, Clarkson University, Potsdam, NY, United States; ^2^Department of Mathematics and Center for Quantitative Biology, Rutgers University, Piscataway, NJ, United States; ^3^Department of Electrical and Computer Engineering, Department of Bioengineering, Northeastern University, Boston, MA, United States; ^4^Laboratory of Systems Pharmacology, Program in Therapeutic Science, Harvard Medical School, Boston, MA, United States

**Keywords:** drug resistance, chemotherapy, phenotype, optimal control theory, singular controls

## Abstract

One of the most important factors limiting the success of chemotherapy in cancer treatment is the phenomenon of drug resistance. We have recently introduced a framework for quantifying the effects of induced and non-induced resistance to cancer chemotherapy (Greene et al., 2018a, 2019). In this work, we expound on the details relating to an optimal control problem outlined in Greene et al. (2018a). The control structure is precisely characterized as a concatenation of bang-bang and path-constrained arcs via the Pontryagin Maximum Principle and differential Lie algebraic techniques. A structural identifiability analysis is also presented, demonstrating that patient-specific parameters may be measured and thus utilized in the design of optimal therapies prior to the commencement of therapy. For completeness, a detailed analysis of existence results is also included.

## 1. Introduction

The ability of cancer chemotherapies to successfully eradicate cancer populations is limited by the presence of drug resistance. Cells may become resistant through a variety of cellular and micro-environmental mechanisms (Gottesman, 2002). These mechanisms are exceedingly complex and diverse, and remain to be completely understood. Equally complex is the manner in which cancer cells obtain the resistant phenotype. Classically resistance was understood to be conferred by random genetic mutations; more recently, the role of epigenetic phenotype switching was discovered as another mediator of resistance (Pisco et al., 2013). Importantly, both of these phenomena were seen as drug-independent, so that the generation of resistance is functionally separate from the selection mechanism (e.g., the drug). However, experimental studies from the past ten years suggest that drug resistance in cancer may actually be induced by the application of chemotherapy (Sharma et al., 2010; Pisco et al., 2013; Goldman et al., 2015; Doherty et al., 2016; Shaffer et al., 2017).

In view of the multitude of ways by which a cancer cell may become chemoresistant, we have previously introduced a mathematical framework to differentiate drug-independent from drug-dependent resistance (Greene et al., 2019). In that work, we demonstrated that induced resistance may play a crucial role in therapy outcome, and also discussed methods by which a treatment's induction potential may be identified via biological assays. An extension of the work was outlined in the conference paper (Greene et al., 2018a), where a formal optimal control problem was introduced and an initial mathematical analysis was performed. The aim of this work is to formalize the parameter identifiability properties of our theoretical model, to establish the existence of the optimal control introduced in Greene et al. (2018a), and to precisely classify the optimal control structure utilizing the Pontryagin Maximum Principle and differential-geometric techniques. A numerical investigation of both the control structure and corresponding objective is also undertaken as a function of patient-specific parameters, and clinical conclusions are emphasized.

The work is organized as follows. In section 2, we briefly review the mathematical model together with the underlying assumptions. Section 3 restates the optimal control problem, and the Maximum Principle is analyzed in section 4. A precise theoretical characterization of the optimal control structure is summarized in section 5. In section 6, we compare theoretical results with numerical computations, and investigate the variation in control structure and objective as a function of parameters. Conclusions are presented in section 8. We also include additional properties, including details on structural identifiability and existence of optimal controls, in Section 7.

## 2. Mathematical Modeling of Induced Drug Resistance

We briefly review the model presented in Greene et al. (2019) and analyzed further in Greene et al. (2018a). In that work, we have constructed a simple dynamical model which describes the evolution of drug resistance through both drug-independent (e.g., random point mutations, gene amplification, stochastic state switching) and drug-dependent (e.g., mutagenicity, epigenetic modifications) mechanisms. Drug-induced resistance, although experimentally observed, remains poorly understood. It is our hope that a mathematical analysis will provide mechanistic insight and produce a more complete understanding of this process by which cancer cells inhibit treatment efficacy.

**Figure 1:**
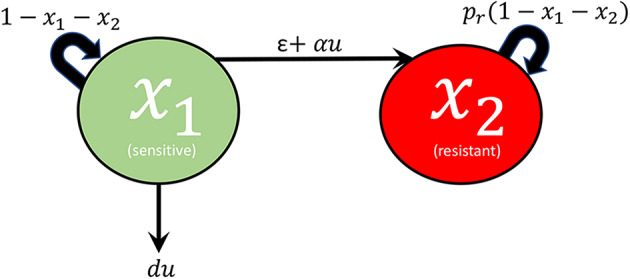
Visualization of interactions considered in system (1).

A network diagram of the model under consideration is provided in Figure 1. Specifically, we assume that the tumor being studied is composed of two types of cells: sensitive (*x*_1_) and resistant (*x*_2_). For simplicity, the drug is taken as completely ineffective against the resistant population, while the log-kill hypothesis (Traina and Norton, 2011) is assumed for the sensitive cells. Complete resistance is of course unrealistic, but can serve as a reasonable approximation, especially when toxicity constraints may limit the total amount of drug that may be administered. Furthermore, this assumption permits a natural metric on treatment efficacy that may not exist otherwise (see section 3). The effect of treatment is considered as a control agent *u*(*t*), which we assume is a locally bounded Lebesgue measurable function taking values in R_+_. Here *u*(*t*) is directly related to the applied drug dosage *D*(*t*), and in the present work we assume that we have explicit control over *u*(*t*). Later, during the formulation of the optimal control problem (section 3), we will make precise specifications on the control set *U*. Even though an arbitrary dosage schedule is unrealistic as a treatment strategy, our objective in this work is to understand the fundamental mathematical questions associated with drug-induced resistance, so we believe the simplification is justified. Furthermore, our results in section 5 suggest that the applied optimal treatment should take a relatively simple form, which may be approximated with sufficient accuracy in a clinical setting. Sensitive and resistant cells are assumed to compete for resources in the tumor microenvironment; this is modeled via a joint carrying capacity, which we have scaled to one. Furthermore, cells are allowed to transition between the two phenotypes in both a drug-independent and drug-dependent manner. All random transitions to the resistant phenotype are modeled utilizing a common term, ϵ*x*_1_, which accounts for both genetic mutations and epigenetic events occurring independently of the application of treatment. Drug-induced deaths are assumed of the form *du*(*t*)*x*_1_, where *d* is the drug cytotoxicity parameter relating to the log-kill hypothesis. Drug-induced transitions are assumed to be of the form α*u*(*t*)*x*_1_, which implies that the per-capita drug-induced transition rate is directly proportional to the dosage [as we assume full control on *u*(*t*), i.e. pharmacokinetics are ignored]. Of course, other functional relationships may exist, but since the problem is not well-studied, we consider it reasonable to begin our analysis in this simple framework. The above assumptions then yield the following system of ordinary differential equations (ODEs):

(1) $$\begin{array}{l} \frac{dx_{1}}{dt}=\left(1-\left(x_{1}+x_{2}\right)\right)x_{1}-\left(\epsilon +\alpha u\left(t\right)\right)x_{1}-du\left(t\right)x_{1}\\ \frac{dx_{2}}{dt}=p_{r}\left(1-\left(x_{1}+x_{2}\right)\right)x_{2}+\left(\epsilon +\alpha u\left(t\right)\right)x_{1}. \end{array}$$

All parameters are taken as non-negative, and 0 ≤ *p*_*r*_ < 1. The restriction on *p*_*r*_ emerges due to (1) already being non-dimensionalized, as *p*_*r*_ represents the relative growth rate of the resistant population with respect to that of the sensitive cells. The condition *p*_*r*_ < 1 thus assumes that the resistant cells divide more slowly than their sensitive counterparts, which is observed experimentally (Shackney et al., 1978; Lee, 1993; Brimacombe et al., 2009). As mentioned previously, many simplifying assumptions are made in system (1). Specifically, both types of resistance (random genetic and epigenetic) are modeled as dynamically equivalent; both possess the same division rate *p*_*r*_ and spontaneous (i.e., drug-independent) transition rate ϵ. Thus, the resistant compartment *x*_2_ denotes the total resistant subpopulation.

The region

(2) $$\begin{array}{l} \Omega =\left\{\left(x_{1},x_{2}\right)|0\leq x_{1}+x_{2}\leq 1,x_{1},x_{2}\geq 0\right\} \end{array}$$

in the first quadrant is forward invariant for any locally bounded Lebesgue measurable treatment function *u*(*t*) taking values in R_+_. Furthermore, if ϵ > 0, the population of (1) becomes asymptotically resistant:

(3) $$\left(\begin{array}{l} x_{1}(t)\\ x_{2}(t) \end{array}\right)\overset{t\to \infty }{\to }\left(\begin{array}{l} 0\\ 1 \end{array}\right).$$

For a proof, see Theorem 2 in SI A in Greene et al. (2019). Thus in our model, the phenomenon of drug resistance is inevitable. However, we may still implement control strategies which, for example, may increase patient survival time. Such aspects will inform the objective introduced in section 3. For more details on the formulation and dynamics of system (1), we refer the reader to Greene et al. (2019).

## 3. Optimal Control Formulation

As discussed in section 2, all treatment strategies *u*(*t*) result in an entirely resistant tumor: $\bar{x}:=\left({\bar{x}}_{1},{\bar{x}}_{2}\right)=\left(0,1\right)$ is globally asymptotically stable for all initial conditions in region Ω. Thus, any chemotherapeutic protocol will eventually fail, and a new drug must be introduced (not modeled in this work, but the subject of future study). Therefore, selecting an objective which minimizes tumor volume (*x*_1_ + *x*_2_) or resistant fraction [*x*_2_/(*x*_1_ + *x*_2_)] at a fixed time horizon would be specious for our modeling framework. However, one can still combine therapeutic efficacy and clonal competition to influence transient dynamics and possibly prolong patient life, as has been shown clinically utilizing real-time patient data (Gatenby et al., 2009).

Toxicity as well as pharmacokinetic constraints limit the amount of drug to be applied at any given instant. Thus, we assume that there exists some number *M* > 0 such that *u*(*t*) ≤ *M* for all *t* ≥ 0. Any Lebesgue measurable treatment regime *u*(*t*) is considered, so that the control set is *U* = [0, *M*] and the set of admissible controls U is

(4) $$\begin{array}{l} U=\left\{u:\left[0,\infty \right)\to \left[0,M\right]|u\text{ is Lebesgue measurable}\right\}. \end{array}$$

Recall that all cellular populations have been normalized to remain in [0, 1]. We assume that there is a *critical tumor volume* *V*_*c*_, at which treatment, by definition, has failed. Our interpretation is that a tumor volume larger than *V*_*c*_ interferes with normal biological function, while *x*_1_ + *x*_2_ ≤ *V*_*c*_ indicates a clinically acceptable state. Different diseases will have different *V*_*c*_ values. For technical reasons needed in section 5 we assume that *V*_*c*_ < 1 − ϵ. This is a mild assumption, since genetic mutation rates ϵ are generally small (Loeb et al., 1974), and our interest is on the impact of induced resistance. Thus

(5) $$\begin{array}{l} V_{c}\in \left(0,1-\epsilon \right). \end{array}$$

Define *t*_*c*_ as the time at which the tumor increases above size *V*_*c*_ for the first time. To be precise,

(6) $$\begin{array}{l} t_{c}\left(u\right):=\max\left\{T|x_{1}\left(t\right)+x_{2}\left(t\right)\leq V_{c}\text{ for all }t\in \left[0,T\right]\right\}. \end{array}$$

Since all treatments approach the state (0, 1), *t*_*c*_(*u*) is well-defined for each treatment *u*(*t*). For simplicity, denote *t*_*c*_ = *t*_*c*_(*u*) in the remainder of the work. The time *t*_*c*_ is then a measure of treatment efficacy, and our goal is then to find those controls u_*_ which maximize *t*_*c*_. Writing in standard form as a minimization problem, we have the following objective:

(7) $$\underset{u\in U}{\min}\{J(u)\}=\underset{u\in U}{\min}\left\{-\int_{0}^{t_{c}}1\;dt\right\}.$$

We are thus seeking a control $u_{*}\left(t\right)\in U$ which *maximizes* *t*_*c*_, i.e. solves the time-optimal minimization problem (7) restricted to the dynamic state equations given by the system described in (1) and the condition *x*_1_(*t*) + *x*_2_(*t*) ≤ *V*_*c*_ for all 0 ≤ *t* ≤ *t*_*c*_. Note that the above is formulated (using the negative sign) as a *minimization* problem to be consistent with previous literature and results related to the Pontryagin Maximum Principle (PMP) (Ledzewicz and Schättler, 2012). Note that maximization is still utilized in section 7.2 and section 4.1, and we believe that the objective will be clear from context. To be consistent with notation utilized later, we denote the system (1) as

(8) $$\begin{array}{l} \dot{x}=f\left(x\right)+u\left(t\right)g\left(x\right), \end{array}$$

where

(9) $$\begin{array}{l} f\left(x\right)=\left(\begin{array}{c} \left(1-\left(x_{1}+x_{2}\right)\right)x_{1}-\epsilon x_{1}\\ p_{r}\left(1-\left(x_{1}+x_{2}\right)\right)x_{2}+\epsilon x_{1} \end{array}\right), \end{array}$$

(10) $$\begin{array}{l} g\left(x\right)=\left(\begin{array}{c} -\left(\alpha +d\right)\\ \alpha \end{array}\right)x_{1} \end{array}$$

and *x*(*t*) = (*x*_1_(*t*), *x*_2_(*t*)). By continuity of solutions, the time *t*_*c*_ must satisfy the terminal condition (*t*_*c*_, *x*(*t*_*c*_)) ∈ *N*, where *N* is the line *x*_1_ + *x*_2_ = *V*_*c*_ in Ω, i.e.,

(11) $$\begin{array}{l} N=\psi^{-1}\left(0\right)\cap \Omega , \end{array}$$

where

(12) $$\begin{array}{l} \psi \left(x_{1},x_{2}\right):=x_{1}+x_{2}-V_{c}. \end{array}$$

With this notation, the path-constraint

(13) $$\begin{array}{l} \psi \left(x_{1}\left(t\right),x_{2}\left(t\right)\right)\leq 0 \end{array}$$

must also hold for 0 ≤ *t* ≤ *t*_*c*_. Equation (13) ensures that the tumor remains below critical volume *V*_*c*_ for the duration of treatment. Equivalently, the dynamics are restricted to lie in the set Ω_*c*_ ⊆ Ω, where

(14) $$\begin{array}{l} \Omega_{c}:=\left\{\left(x_{1},x_{2}\right)|0\leq x_{1}+x_{2}\leq V_{c},x_{1},x_{2}\geq 0\right\}, \end{array}$$

for all times *t* such that *t* ∈ [0, *t*_*c*_]. The initial state

(15) $$\begin{array}{l} x_{0}=\left(x_{1}^{0},x_{2}^{0}\right) \end{array}$$

is also assumed to lie in Ω_*c*_. Except for section 7.1 where we restrict to the case $x_{2}^{0}=0$, the remainder of the work allows for arbitrary $x_{2}^{0}\in \left[0,V_{c}\right)$.

## 4. Maximum Principle

We dedicate the present section to characterize the optimal control utilizing the Pontryagin Maximum Principle (PMP). The subsequent analysis is strongly influenced by the Lie-derivative techniques introduced by Sussmann (1982, 1987a,b,c). For an excellent source on both the general theory and applications to cancer biology, see the textbooks by Ledzewicz and Schättler (2012) and Schättler and Ledzewicz (2015).

Before starting our analysis of the behavior and response of system (1) to applied treatment strategies *u*(*t*) utilizing geometric methods, we would like to mention that we have not found a reference for existence of optimal controls for a problem such as this, due perhaps to the non-standard character of it (maximization of time, path constraints). For this reason, we have added a self-contained proof of regarding existence in section 7.2.

### 4.1. Elimination of Path Constraints

We begin our analysis by separating interior controls from those determined by the path-constraint (13) (equivalently, *x* ∈ *N*). The following theorem implies that outside of the one-dimensional manifold *N*, the optimal pair (x_*_, u_*_) solves the same local optimization problem without the path and terminal constraints. More precisely, the necessary conditions of the PMP (see section 4.2) at states not on *N* are exactly the conditions of the corresponding maximization problem with no path or terminal constraints.

**THEOREM 1**. Suppose that x_*_ *is an optimal trajectory. Let t*_1_ be the first time such that x_*_(*t*) ∈ *N*. *Fix* δ > 0 *such that t*_1_ − δ > 0, *and*

(16) $$\begin{array}{l} \xi =x_{*}\left(t_{1}-\delta \right). \end{array}$$

*Define z*(*t*): = x_*_(*t*)|_*t* ∈ [0,_*t*__1_−δ]_. *Then the trajectory z is a local solution of the corresponding time maximization problem t*_*c*_ *with boundary conditions x*(0) = *x*^0^, *x*(*t*_c_) = ξ, *and no additional path constraints. Hence at all times t, the path z (together with the corresponding control and adjoint) must satisfy the corresponding unconstrained Pontryagin Maximum Principle*.

*Proof*. We first claim that *z* satisfies the path-constrained maximization problem with boundary conditions $x\left(0\right)=x^{0},x\left(t_{c}\right)=\xi$. This is a standard dynamic programming argument: if there exists a trajectory $\bar{z}$ such that $\bar{z}\left(\tau \right)=\xi$, τ > *t*_1_ − δ, concatenate $\bar{z}\left(t\right){|}_{t\in \left[0,\tau \right]}$ with x_*_(*t*)|_*t* ∈ [τ,*t*_*c*_]_ at *t* = τ to obtain a feasible trajectory satisfying all constraints. This trajectory then has total time τ + δ + *t*_*c*_ − *t*_1_ > *t*_*c*_, contradicting the global optimality of x_*_.

Recall that *t*_1_ was the first time such that x_*_(*t*) ∈ *N*. Since *z* is compact, we can find a neighborhood of *z* that lies entirely in {*x*|*x* ∉ *N*}. As the Maximum Principle is a local condition with respect to the state, this completes the proof.     □

Theorem 1 then tells us that for states *x* = (*x*_1_, *x*_2_) such that *x*_1_ + *x*_2_ < *V*_*c*_, the corresponding unconstrained PMP must be satisfied by any extremal lift of the original problem. (Recall that an extremal lift of an optimal trajectory is obtained by adding the Lagrange multipliers, or adjoint variables, to the control and state; see details in Definition 2.2.4, page 95, Chapter 2 of Ledzewicz and Schättler, 2012). We have now demonstrated that the optimal control consists of concatenations of controls obtained from the unconstrained necessary conditions and controls of the form (18). In the next section, we analyze the Maximum Principle in the region *x*_1_ + *x*_2_ < *V*_*c*_. Furthermore, the constraint (13) has generic order one. In other words,

(17) $$\begin{array}{l} L_{g}\psi =\nabla \psi \cdot g\neq 0. \end{array}$$

Therefore, the feedback control (also known as the constrained control) can be found by differentiating the function (12) to insure that trajectories remain on the line *N*:

(18) $$\begin{array}{l} u_{p}\left(x_{1},x_{2}\right)=\frac{1}{d}\frac{\left(1-\left(x_{1}+x_{2}\right)\right)\left(x_{1}+p_{r}x_{2}\right)}{x_{1}}. \end{array}$$

**Figure 2:**
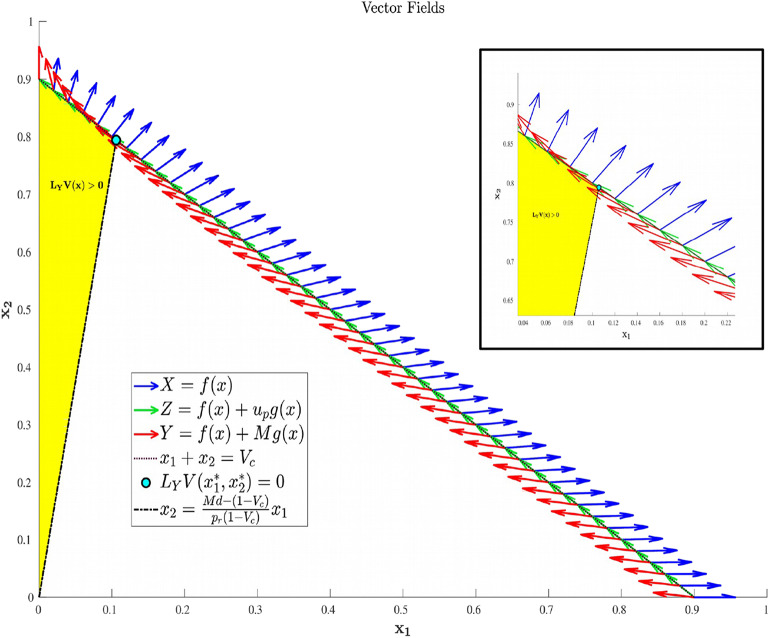
Region in Ω_*c*_ where *L*_*Y*_*V*(*x*) is guaranteed to be positive. That is, applying the maximal dosage *M* results in an increasing cancer population in the yellow-shaded region of phase-space.

Its existence however does not imply its feasibility, which is discussed below. Specifically, *u*_*p*_ can be shown to be a decreasing function of *x*_1_ which is feasible on the portion of *N* satisfying $x_{1}^{*}\leq x_{1}\leq V_{c}$, where $x_{1}^{*}$ is given in (20), and infeasible elsewhere. This is proven in Proposition 3, and the geometric structure is depicted in Figure 2. Propositions 4 and 5 provide characterizations of the volume dynamics in certain regions of phase space, and are included here for completeness.

**Proposition 2**. *Suppose that the maximal dosage M satisfies*

(19) $$\begin{array}{l} M>\frac{\left(1-V_{c}\right)\left(1-p_{r}\right)}{d}. \end{array}$$

*and the point* $x^{*}=\left(x_{1}^{*},x_{2}^{*}\right)\in N$ *with coordinates*

(20) $$\begin{array}{l} x_{1}^{*}=\frac{p_{r}\left(1-V_{c}\right)V_{c}}{dM-\left(1-V_{c}\right)\left(1-p_{r}\right)},\\ x_{2}^{*}=V_{c}\left(1-\frac{p_{r}\left(1-V_{c}\right)}{dM-\left(1-V_{c}\right)\left(1-p_{r}\right)}\right). \end{array}$$

*Denote by Y*(*x*) = *f*(*x*) + *Mg*(*x*) *the vector field corresponding to the maximal allowed dosage M* [*here, f and g are the functions defined in* (*9*), (*10*)]. *The Lie derivative, for any x* ∈ *N, of the volume function V*(*x*) = *x*_1_ + *x*_2_ *with respect to Y is*

- *positive if* $x_{1}<x_{1}^{*}$,
- *zero at* $\left(x_{1}^{*},x_{2}^{*}\right)$, *and*
- *negative if* $x_{1}>x_{1}^{*}$.

*Proof*. We verify the above claims with a direct calculation. Let *L*_*Y*_*V*(*x*) denotes the Lie derivative of *V*(*x*) with respect to *Y*. Thus, for *x* ∈ *N*,

$$\begin{array}{l} L_{Y}V\left(x\right)=\nabla V\left(x\right)\cdot Y\left(x\right)\\ \;\\ \;=\left(\begin{array}{l} 1\\ 1 \end{array}\right)\cdot \left(\begin{array}{c} \left[1-V_{c}-\epsilon -\left(\alpha +d\right)M\right]x_{1}\\ \left[\epsilon +\alpha M-p_{r}\left(1-V_{c}\right)\right]x_{1}+p_{r}\left(1-V_{c}\right)V_{c} \end{array}\right)\\ \;=\left[1-V_{c}-\epsilon -\left(\alpha +d\right)M\right]x_{1}\\ \;+\left[\epsilon +\alpha M-p_{r}\left(1-V_{c}\right)\right]x_{1}\\ \;+p_{r}\left(1-V_{c}\right)V_{c}\\ \;\\ \;=\left[\left(1-V_{c}\right)\left(1-p_{r}\right)-dM\right]x_{1}+p_{r}\left(1-V_{c}\right)V_{c}. \end{array}$$

Assuming $M>\frac{\left(1-V_{c}\right)\left(1-p_{r}\right)}{d}$, the sign of *L*_*Y*_*V*(*x*) is as in the statement of the proposition.     □

Proposition 2 implies that if the allowable dosage is large enough (Equation 9), treatment can at least decrease the tumor in certain regions of phase space. If this condition was not met, then the applied drug would generally be ineffective in reducing the tumor volume *V*, and hence not be utilized in a clinical scenario.

**Proposition 3**. *Let *x* be a point on the line *N*. The feedback control *u*_*p*_ is unfeasible if* $x_{1}\in \left(0,x_{1}^{*}\right)$, *and is feasible if* $x_{1}\in \left(x_{1}^{*},V_{c}\right)$

*Proof*. For *x* ∈ *N* we compute

$$\begin{array}{l} u_{p}\left(x\right)=\frac{\left(1-V_{c}\right)\left(1-p_{r}\right)}{d}+\frac{\left(1-V_{c}\right)p_{r}V_{c}}{dx_{1}}\geq 0. \end{array}$$

It is straightforward to check that *u*_*p*_ > *M* if $x_{1}<x_{1}^{*}$. In addition, the feedback control, when restricted to points in *N*, is a decreasing function with respect to *x*_1_. Thus, it is feasible for *x* ∈ *N* if $x_{1}\in \left(x_{1}^{*},V_{c}\right)$.     □

**Proposition 4**. *For x* = (*x*_1_, *x*_2_) ∈ Ω_*c*_ *with*

(21) $$\begin{array}{l} x_{2}>\frac{dM-\left(1-V_{c}\right)}{p_{r}\left(1-V_{c}\right)}x_{1}, \end{array}$$

*the Lie derivative *L*_*Y*_*V*(*x*) is positive*.

*Proof*. As in Proposition 2, we compute *L*_*Y*_*V*(*x*) directly:

$$\begin{array}{l} L_{Y}V\left(x\right)=\left(1-\left(x_{1}+x_{2}\right)\right)\left(x_{1}+p_{r}x_{2}\right)-dMx_{1}\\ \;\geq \left(1-V_{c}\right)\left(x_{1}+p_{r}x_{2}\right)-dMx_{1}\\ \;=\left[\left(1-V_{c}\right)-dM\right]x_{1}+p_{r}\left(1-V_{c}\right)x_{2}\\ \;>\left[\left(1-V_{c}\right)-dM\right]x_{1}+p_{r}\left(1-V_{c}\right)\frac{dM-\left(1-V_{c}\right)}{p_{r}\left(1-V_{c}\right)}x_{1}\\ \;=0, \end{array}$$

where the first inequality utilizes *V* ≤ *V*_*c*_, and the second relies on (21)     □

**Proposition 5**. *For*

$$\begin{array}{l} M>\frac{1-\epsilon }{\alpha +d}, \end{array}$$

*trajectories corresponding to the maximal dosage M have a decreasing sensitive cellular population*.

*Proof*. For *u*(*t*) ≡ *M*, the corresponding sensitive trajectory is given by

$$\begin{array}{l} \dot{x_{1}}=\left(1-\left(x_{1}+x_{2}\right)\right)x_{1}-\epsilon x_{1}-\left(\alpha +d\right)Mx_{1}\\ \;<\left(1-\left(x_{1}+x_{2}\right)\right)x_{1}-\epsilon x_{1}-\left(1-\epsilon \right)x_{1}\\ \;=-\left(x_{1}+x_{2}\right)x_{2}\leq 0 \end{array}$$

Note that we are assuming here that the maximal dosage *M* satisfies $M>\frac{1-\epsilon }{\alpha +d}$.     □

### 4.2. Maximum Principle and Necessary Conditions at Interior Points

Necessary conditions for the optimization problem discussed in section 3 without path or terminal constraints are derived from the Pontryagin Maximum Principle (Pontryagin, 1987; Ledzewicz and Schättler, 2012). The corresponding Hamiltonian function *H* is defined as

(22) $$\begin{array}{l} H\left(\lambda_{0},\lambda ,x,u\right)=-\lambda_{0}+\langle \lambda ,f\left(x\right)\rangle +u\Phi \left(x,\lambda \right), \end{array}$$

where λ_0_ ≥ 0 and λ ∈ R^2^. Here 〈·, ·〉 denotes the standard inner product on R^2^ and, since the dynamics are affine in the control *u*, Φ(*x*, λ) is the switching function:

(23) $$\begin{array}{l} \Phi \left(x,\lambda \right)=\langle \lambda ,g\left(x\right)\rangle . \end{array}$$

The Maximum Principle then yields the following theorem:

**THEOREM 6**. *If the extremal* (x_*_, u_*_) *is optimal, there exists* λ_0_ ≥ 0 *and a covector* (*adjoint*) $\lambda :\left[0,t_{c}\right]\to {\left({\text{R}}^{2}\right)}^{*}$, *such that the following hold*:

1. (λ_0_, λ(*t*)) ≠ 0 *for all* *t* ∈ [0, *t*_*c*_].
2. λ(*t*) = (λ_1_(*t*), λ_2_(*t*)) *satisfies the second-order differential equation*
  (24) $$\begin{array}{l} \begin{array}{l} \dot{\lambda }\left(t\right)=\left(\begin{array}{cc} 2x_{1}+x_{2}+\epsilon -1 & p_{r}x_{2}-\epsilon \\ x_{1} & p_{r}\left(2x_{2}+x_{1}-1\right) \end{array}\right)\lambda \left(t\right)\\ \;+u\left(t\right)\left(\begin{array}{cc} \alpha +d & -\alpha \\ 0 & 0 \end{array}\right)\lambda \left(t\right) \end{array} \end{array}$$
3. u_*_(*t*) *minimizes* *H* *pointwise over the control set* *U*:
  $$\begin{array}{l} H\left(\lambda_{0},\lambda ,x_{*}\left(t\right),u_{*}\left(t\right)\right)=\underset{v\in U}{\min}H\left(\lambda_{0},\lambda ,x_{*}\left(t\right),v\right). \end{array}$$
  *Thus, the control* u_*_(*t*) *must satisfy*
  (25) $$u_{*}(t)=\{\begin{array}{ll} 0 & \Phi (t)>0,\\ M & \Phi (t)<0. \end{array}$$
  *where*
  (26) $$\begin{array}{l} \Phi \left(t\right):=\Phi \left(x_{*}\left(t\right),\lambda \left(t\right)\right). \end{array}$$
4. *The Hamiltonian* *H* *is identically zero along the extremal lift* (x_*_(*t*), u_*_(*t*), λ(*t*)):
  (27) $$\begin{array}{l} H\left(\lambda_{0},\lambda \left(t\right),x_{*}\left(t\right),u_{*}\left(t\right)\right)\equiv 0. \end{array}$$

*Proof*. Most statements of Theorem 6 follow directly from the Maximum Principle, so proofs are omitted. In particular, items (1), (2), and the first part of (3) are immediate consequences (Ledzewicz and Schättler, 2012). Equation (25) follows directly since we minimize the function *H*, which is affine in *u* (see Equation 22). The Hamiltonian vanishes along (x_*_(*t*), u_*_(*t*), λ(*t*)) since it is independent of an explicit time *t* dependence and the final time *t*_*c*_ is free, the latter being a consequence of the transversality condition.     □

**Proposition 7**. *For all t* ∈ [0, *t*_*c*_], *the adjoint* λ(*t*) *corresponding to the extremal lift* (x_*_(*t*), u_*_(*t*), λ(*t*)) *is nonzero*.

*Proof*. This is a general result relating to free end time problems. We include a proof here for completeness. Suppose that there exists a time *t* ∈ [0, *t*_*c*_] such that λ(*t*) = 0. By (22), the corresponding value of the Hamiltonian is *H*(λ_0_, λ(*t*), x_*_(*t*), u_*_(*t*)) = −λ_0_. By item (4) in Theorem 6, *H* ≡ 0, which implies that λ_0_ = 0. This contradicts item (1) in Theorem 6. Hence, λ(*t*) ≠ 0 on [0, *t*_*c*_].     □

### 4.3. Geometric Properties and Existence of Singular Arcs

We now undertake a geometric analysis of the optimal control problem utilizing the affine structure of system (8) for interior states (i.e., controls which satisfy Theorem 6). We call such controls *interior extremals*, and all extremals in this section are assumed to be interior. The following results depend on the independence of the vector fields *f* and *g*, which we use to both classify the control structure for abnormal extremal lifts (extremal lifts with λ_0_ = 0), as well as characterize the switching function dynamics via the Lie bracket.

**Proposition 8**. *For all x*_1_ ∈ Ω, *x*_1_ > 0, *the vector fields f*(*x*) *and g*(*x*) *are linearly independent*.

*Proof*. Define *A*(*x*) = *A*(*x*_1_, *x*_2_) to be the matrix

(28) $$\begin{array}{l} \begin{array}{l} A\left(x\right)=\left(f\left(x\right)g\left(x\right)\right)\\ \;=\left(\begin{array}{cc} \left(1-\left(x_{1}+x_{2}\right)-\epsilon \right)x_{1} & -\left(\alpha +d\right)x_{1}\\ p_{r}\left(1-\left(x_{1}+x_{2}\right)\right)x_{2}+\epsilon x_{1} & \alpha x_{1} \end{array}\right). \end{array} \end{array}$$

The determinant of *A* can calculated as

(29) $$\begin{array}{l} \det A\left(x\right)=\alpha x_{1}^{2}\kappa \left(x\right)+p_{r}\left(\alpha +d\right)x_{2}x_{1}\kappa \left(x\right)+\epsilon dx_{1}^{2} \end{array}$$

where

(30) $$\begin{array}{l} \kappa \left(x\right):=1-\left(x_{1}+x_{2}\right). \end{array}$$

As *x*_1_(*t*) + *x*_2_(*t*) ≤ 1 for all *t* ≥ 0, κ(*x*(*t*)) ≥ 0, and we see that det*A*(*x*) = 0 in Ω if and only if *x*_1_ = 0, completing the proof.     □

The line *x*_1_ = 0 is invariant in Ω, and furthermore the dynamics in the set are independent of the control *u*(*t*). Conversely, $x_{1}^{0}>0$ implies that *x*_1_(*t*) > 0 for all *t* ≥ 0. We concern our analysis only in this latter case, and so without loss of generality, **f(x) and g(x) are linearly independent in the region of interest Ω_c_**.

We begin by showing that abnormal extremal lifts are easily characterized. We recall that an extremal lift is abnormal if λ_0_ = 0, i.e., if the Hamiltonian is independent of the objective.

**THEOREM 9**. *Abnormal extremal lifts at interior points, i.e., extremal lifts corresponding to* λ_0_ = 0, *are constant and given by the maximal* (*M*) *or minimal* (0) *dosage*.

*Proof*. Assume that u_*_ switches values at some time *t*. From (25), we must have that Φ(*t*) = 0. Since λ_0_ = 0 and Φ(*t*) = 〈λ(*t*), *g*(x_*_(*t*))〉, Equation (22) reduces to

(31) $$\begin{array}{l} H\left(t\right)=\langle \lambda \left(t\right),f\left(x_{*}\left(t\right)\right)\rangle =0. \end{array}$$

Thus, λ(*t*) is orthogonal to both *f*(x_*_(*t*)) and *g*(x_*_(*t*)). Since *f* and *g* are linearly independent (Proposition 8), this implies that λ(*t*) = 0. But this contradicts Proposition 7. Hence, no such time *t* exists, and u_*_(*t*) is constant. The constant sign of Φ thus corresponds to *u* = 0 or *u* = *M* (see Equation 25).     □

The control structure for abnormal extremal lifts is then completely understood via Theorem 9. To analyze the corresponding behavior for normal extremal lifts, without loss of generality we assume that λ_0_ = 1. Indeed, λ(*t*) may be rescaled by λ_0_ > 0 to yield an equivalent version of Theorem 6. We thus assume that the Hamiltonian *H*(*t*) evaluated along (λ(*t*), x_*_(*t*), u_*_(*t*)) is of the form

(32) $$\begin{array}{l} H\left(t\right)=-1+\langle \lambda \left(t\right),f\left(x_{*}\left(t\right)\right)\rangle +u_{*}\left(t\right)\Phi \left(t\right)\equiv 0. \end{array}$$

We recall the Lie bracket as the first-order differential operator between two vector fields *X*_1_ and *X*_2_:

(33) $$\begin{array}{l} \left[X_{1},X_{2}\right]\left(z\right)=DX_{2}\left(z\right)X_{1}\left(z\right)-DX_{1}\left(z\right)X_{2}\left(z\right), \end{array}$$

where, for example, *DX*_2_(*z*) denotes the Jacobian of *X*_2_ evaluated at *z*. As *f* and *g* are linearly independent in Ω, there exist γ, β ∈ *C*^∞^(Ω) such that

(34) $$\begin{array}{l} \left[f,g\right]\left(x\right)=\gamma \left(x\right)f\left(x\right)+\beta \left(x\right)g\left(x\right), \end{array}$$

for all *x* ∈ Ω. Explicitly, we compute γ and β:

(35) $$\begin{array}{l} \gamma \left(x\right)=-\frac{\left(\alpha +d\right)x_{1}^{2}}{\det A\left(x\right)}\left(ax_{1}+bx_{2}-c\right), \end{array}$$

(36) $$\begin{array}{l} \beta (x)=\frac{x_{1}^{2}}{\det A(x)}(\alpha (1-p_{r})\kappa (x)(\kappa (x)-\epsilon )+\epsilon d(x_{1}+p_{r}x_{2}\\ \;+\kappa (x)-\epsilon )), \end{array}$$

where

(37) $$\begin{array}{l} a=\alpha \left(\left(1-p_{r}\right)+\frac{d}{\alpha +d}\right), \end{array}$$

(38) $$\begin{array}{l} b=\alpha \left(1-p_{r}\right)+dp_{r}, \end{array}$$

(39) $$\begin{array}{l} c=\alpha \left(1-p_{r}\right)+\epsilon d. \end{array}$$

Clearly, for parameter values of interest (recall 0 < *p*_*r*_ < 1), *a, b, c* > 0. The assumption (5) guarantees that β(*x*) > 0 on 0 < *x*_1_ + *x*_2_ < *V*_*c*_.

From (25), the sign of the switching function Φ determines the value of the control u_*_. As λ and x_*_ are solutions of differential equations, Φ is differentiable. The dynamics of Φ can be understood in terms of the Lie bracket [*f, g*]:

(40) $$\begin{array}{l} \dot{\Phi }\left(t\right)=\frac{d}{dt}\langle \lambda \left(t\right),g\left(x_{*}\left(t\right)\right)\rangle \end{array}$$

(41) $$\begin{array}{l} =\gamma \left(x_{*}\left(t\right)\right)\langle \lambda \left(t\right),f\left(x_{*}\left(t\right)\right)\rangle +\beta \left(x_{*}\left(t\right)\right)\Phi \left(t\right). \end{array}$$

The last lines of the above follow from (34) as well as the linearity of the inner product. We are then able to derive an ODE system for x_*_ and Φ. Equation (32) allows us to solve for 〈λ(*t*), *f*(x_*_(*t*))〉:

(42) $$\begin{array}{l} \langle \lambda \left(t\right),f\left(x_{*}\left(t\right)\right)\rangle =1-u_{*}\left(t\right)\Phi \left(t\right). \end{array}$$

Substituting the above into (41) then yields the following ODE for Φ(*t*), which we view as coupled to system (8) via (25):

(43) $$\dot{\Phi }(t)\;=\gamma (x_{*}(t))+\left(\beta (x_{*}(t))-u_{*}(t)\gamma (x_{*}(t))\right)\Phi (t).$$

The structure of the optimal control at interior points may now be characterized as a combination of bang-bang and singular arcs. We recall that the control (or, more precisely, the extremal lift) u_*_ is singular on an open interval *I* ⊂ [0, *t*_*c*_] if the switching function Φ(*t*) and all its derivatives are identically zero on *I*. On such intervals, Equation (25) does not determine the value of u_*_, and a more thorough analysis of the zero set of Φ(*t*) is necessary. Indeed, for a problem such as ours, aside from controls determined by the path constraint ψ(*x*_1_(*t*), *x*_2_(*t*)) ≤ 0, singular arcs are the only candidates for optimal controls that may take values outside of the set {0, *M*}. Conversely, times *t* where Φ(*t*) = 0 but Φ^(*n*)^(*t*) ≠ 0 for some *n* ≥ 1 denote candidate bang-bang junctions, where the control may switch between the vertices 0 and *M* of the control set *U*. Note that the parity of the smallest such *n* determines whether a switch actually occurs: *n* odd implies a switch, while for *n* even u_*_ remains constant. Equation (43) allows us to completely characterize the regions in the (*x*_1_, *x*_2_) plane where singular arcs are attainable, as demonstrated in the following proposition.

**Proposition 10**. *Singular arcs are only possible in regions of the* (*x*_1_, *x*_2_) *plane where* γ(*x*) = 0. *Furthermore, as x*_1_(*t*) > 0 *for all t* ≥ 0, *the region* {*x* ∈ R^2^ | γ (*x*) = 0} ∩ Ω *is the line*

(44) $$\begin{array}{l} ax_{1}+bx_{2}-c=0, \end{array}$$

*where a, b, c are defined in (37–39)*.

*Proof*. As discussed prior to the statement of Proposition 10, a singular arc must occur on a region where both Φ(*t*) and $\dot{\Phi }\left(t\right)$ are identically zero (as well as all higher-order derivatives). Denoting by x_*_(*t*) the corresponding trajectory in the (*x*_1_, *x*_2_) phase plane, we may calculate $\dot{\Phi }\left(t\right)$ from equation (43):

(45) $$\begin{array}{l} \dot{\Phi }\left(t\right)=\gamma \left(x_{*}\left(t\right)\right). \end{array}$$

Note we have substituted the assumption Φ(*t*) = 0. Clearly we must also have that γ(x_*_(*t*)) = 0, thus implying that $x_{*}\left(t\right)\in \gamma^{-1}\left(0\right)$, as desired. The last statement of the proposition follows immediately from Equation (35).     □

Proposition 10 implies that singular solutions can only occur along the line *ax*_1_ + *bx*_2_ − *c* = 0. Thus, define regions in the first quadrant as follows:

(46) $$\begin{array}{l} \Omega_{c}^{+}:=\left\{x\in \Omega |\gamma \left(x\right)>0\right\}, \end{array}$$

(47) $$\begin{array}{l} \Omega_{c}^{-}:=\left\{x\in \Omega |\gamma \left(x\right)<0\right\}, \end{array}$$

(48) $$\begin{array}{l} L=\left\{x\in \Omega |\gamma \left(x\right)=0\right\}. \end{array}$$

**Figure 3:**
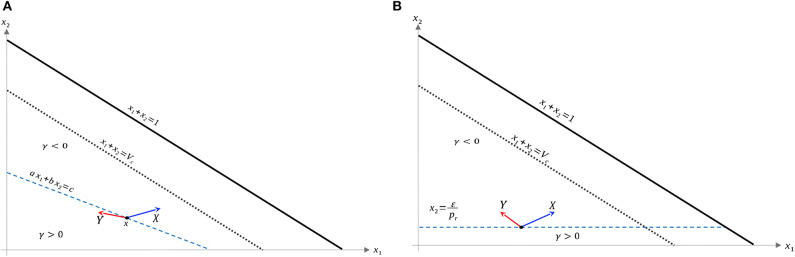
Domain in (*x*_1_, *x*_2_) plane. **(A)** Region where γ changes sign. We see that inside the triangular region *x*_1_ + *x*_2_ ≤ 1 of the first quadrant, γ changes sign only along the line *ax*_1_ + *bx*_2_ − *c* = 0. For this line to be interior to Ω_*c*_ as depicted, we must be in the parameter regime indicated in (49). *X* and *Y* vector fields corresponding to vertices of control set *U*. For singular controls to lie in *U*, *X* and *Y* must point to opposite sides along L. **(B)** Same as in **(A)**, but with α = 0.

Recall that Ω_*c*_ is simply the region in Ω prior to treatment failure, i.e., 0 ≤ *V* ≤ *V*_*c*_, *x*_1_, *x*_2_ ≥ 0. From (35), Ω_*c*_ is partitioned as in Figure 3B. From (35) and (37–39), L is a line with negative slope −*b*/*a*. Furthermore, necessary and sufficient conditions for L to lie interior to Ω_*c*_ are $\frac{c}{a},\frac{c}{b}\leq V_{c}$. From (37)–(39), this occurs if and only if

(49) $$\begin{array}{l} \epsilon \leq \min\{\frac{\alpha }{\alpha +d}-\frac{1-V_{c}}{d}\left(\alpha (1-p_{r})+\frac{\alpha d}{\alpha +d}\right),\\ \;p_{r}-\frac{1-V_{c}}{d}\left(\alpha (1-p_{r})+dp_{r}\right)\}. \end{array}$$

As we have assumed that ϵ is small, and that *V*_*c*_ ≈ 1, this inequality is not restrictive, and we assume it is satisfied for the remainder of the work. We note an important exception below: when α = 0 the inequality is never satisfied with ϵ > 0; for such parameter values, line L is horizontal (Figure 3B). We note that this does not change the qualitative results presented below. Of course, other configurations of the line *ax*_1_ + *bx*_2_ = *c* and hence precise optimal syntheses may exist, but we believe the situation illustrated in Figure 3A is sufficiently generic for present purposes.

With the existence of singular arcs restricted to the line γ = 0 by Proposition 10, we now investigate the feasibility of such solutions. Recall that the treatment *u*(*t*) must lie in the control set *U* = [0, *M*], for some *M* > 0 corresponding to the maximally tolerated applied dosage. Defining the vector field *X*(*x*) and *Y*(*x*) as the vector fields corresponding to the vertices of *U*,

(50) $$\begin{array}{l} \begin{array}{l} X\left(x\right):=f\left(x\right),\\ Y\left(x\right):=f\left(x\right)+Mg\left(x\right), \end{array} \end{array}$$

a singular control takes values in *U* at x∈L if and only if *X*(*x*) and *Y*(*x*) point in different directions along L. More precisely, the corresponding Lie derivatives *L*_*X*_γ(*x*) and *L*_*Y*_γ(*x*) must have opposite signs (see Figure 3A). The following proposition determines parameter values where this occurs.

**Proposition 11**. *Suppose that α* > 0, *so that drug has the potential to induce resistance. Also, let the maximally tolerated dosage M satisfy*

(51) $$\begin{array}{l} M>\frac{\alpha +d}{\alpha (\alpha +d)+\alpha d}(d\left(\frac{\alpha }{\alpha +d}-\epsilon \right)+\epsilon d(p_{r}-\alpha )\\ \;-2\alpha d(1-p_{r})). \end{array}$$

*Then the following hold along* L:

1. *L*_*X*_γ < 0,
2. *L*_*Y*_γ < 0 *as* $\left(x_{1},x_{2}\right)\to \left(0,\frac{c}{b}\right)$ *in* Ω,
3. *L*_*Y*_γ > 0 *at* $\left(x_{1},x_{2}\right)=\left(\frac{c}{a},0\right)$, *and*
4. *L*_*Y*_γ *is monotonically decreasing as a function of x*_1_.

*Thus*, L *contains a segment* $\bar{L}\subset L$ *which is a singular arc. Note that* $\bar{L}$ *is precisely the region in* L *where* *L*_*Y*_γ *is positive*.

*Proof*. The proof is purely computational.     □

Note that if inequality (51) is not satisfied, then singular arcs are not in the domain Ω_*c*_.

**Figure 4:**
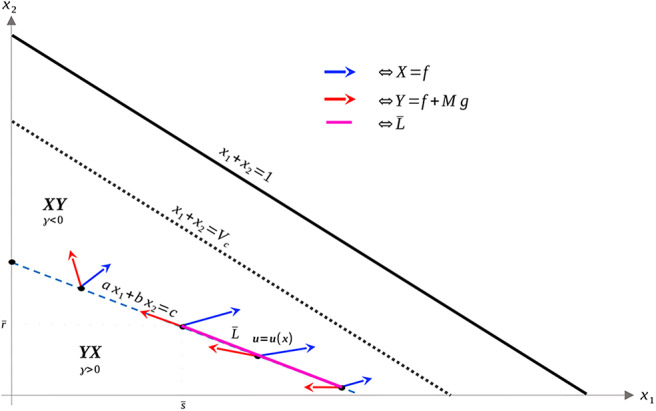
Geometry of vector fields *X* and *Y* with α > 0 and *M* satisfying (51). As in Proposition 11, this can be understood via the corresponding Lie derivatives of γ. Note that near *x*_2_ = 0, *X*, and *Y* point to opposite sides of *L*, while at $\left(x_{1},x_{2}\right)=\left(0,\frac{c}{b}\right)$, both *X* and *Y* point away from γ > 0. The line $\bar{\text{L}}$ is the unique singular arc in Ω_*c*_.

The geometry of Proposition 11 is illustrated in Figure 4. Thus, assuming α > 0 and *M* as in (51), singular arcs exist along the segment $\bar{L}\subset L$. Furthermore, the corresponding control has a unique solution *u*_*s*_, which may be computed explicitly. Indeed, as the solution must remain on the line L, or equivalently, *ax*_1_ + *bx*_2_ = *c*, taking the time derivative of this equation yields *aẋ*_1_ + *bẋ*_2_ = 0, and substituting the expressions (1) we compute *u*_*s*_ as

(52) $$u_{s}(t)=\frac{\left(1-(x_{1}(t)+x_{2}(t))\right)\left(ax_{1}(t)+p_{r}bx_{2}(t)\right)+\epsilon (b-a)x_{1}(t)}{2\alpha (1-p_{r})dx_{2}(t)},$$

where *a, b, c* are given by (37–39) and *x*_2_ and *x*_1_ satisfy *ax*_1_ + *bx*_2_ = *c*. As discussed previously, *x*_1_(*t*) > 0 for $x_{1}^{0}>0$, so this formula is well-defined. Proposition 11 implies that it is possible to simplify Equation (52) as a function of *x*_1_ (i.e. as a *feedback law*) for $x_{1}\in \left(\bar{s},\frac{c}{a}\right)$, for some $\bar{s}>0$, but since its value will not be needed, we do not provide its explicit form. Note that the maximal dose *M* is achieved precisely at $x_{1}=\bar{s}$ where vector field *Y* is parallel to L. Thus, at this $\bar{s}$, the trajectory must leave the singular arc, and enter the region $\Omega_{c}^{-}$. As *ẋ*_2_ ≥ 0, trajectories must follow L in the direction of decreasing *x*_1_ (see Figure 4). We summarize these results in the following theorem.

**THEOREM 12**. *If* α > 0, *and M satisfies* (*51*), *a singular arc exists in the* (*x*_1_, *x*_2_) *plane as a segment of the line* L. *Along this singular arc, the control is given by Equation* (*52*), *where ax*_1_ + *bx*_2_ = *c*. Therefore, in this case the necessary minimum conditions on u_*_ *from* (*25*) *can be updated as follows*:

(53) $$u_{*}(t)=\{\begin{array}{ll} 0 & \Phi (t)>0,\\ M & \Phi (t)<0,\\ u_{s}(t), & \Phi (t)\equiv 0\text{ for }t\in I, \end{array}$$

*where I is an open interval. Recall again that this is the optimal control at points interior to* Ω_*c*_.

*Proof*. See the discussion immediately preceding Theorem 12.     □

In the case α = 0, the line L is horizontal, and as *x*_2_ is increasing, no segment $\bar{L}\subseteq L$ is admissible in phase space. Thus, the interior controls in this case are bang-bang; for a visualization (see Figure 3B).

**THEOREM 13**. *If* α = 0, *there are no singular arcs for the optimal time problem presented in section 3. Thus, the interior control structure is bang-bang*.

Outside of the singular arc $\bar{L}$, the control structure is completely determined by (25) and (43). The precise result, utilized later for the optimal synthesis presented in section 5, is stated in the following theorem. We first introduce a convenient (and standard) notation. Let finite words on *X* and *Y* denote the concatenation of controls corresponding to vector fields *X* (*u* ≡ 0) and *Y* (*u* ≡ *M*), respectively. The order of application is read left-to-right, and an arc appearing in a word may not actually be applied (e.g. *XY* denotes an *X* arc followed by a *Y* arc or a *Y* arc alone).

**THEOREM 14**. *Consider an extremal lift* Γ = ((*x, u*), λ). *Trajectories* *x* *remaining entirely in* $\Omega_{c}^{+}$ *or* $\Omega_{c}^{-}$ *can have at most one switch point. Furthermore, if* $x\in \Omega_{c}^{+}$, *then the corresponding control is of the form YX. Similarly*, $x\in \Omega_{c}^{-}$ *implies that u* = *XY*. *Hence multiple switch points must occur across the singular arc* $\bar{L}$.

*Proof*. If τ is a switching time, so that Φ(τ) = 0, Equation (43) allows us to calculate $\dot{\Phi }\left(\tau \right)$ as

(54) $$\begin{array}{l} \dot{\Phi }\left(\tau \right)=\gamma \left(x\left(\tau \right)\right). \end{array}$$

Thus, in $\Omega_{c}^{+}$ where γ > 0, $\dot{\Phi }\left(\tau \right)>0$, and hence Φ must increase through τ. The expression for the control (25) then implies that a transition from a *Y*-arc to an *X*-arc occurs at τ (i.e., a *YX* arc). Furthermore, another switching time cannot occur unless *x* leaves $\Omega_{c}^{+}$, since otherwise there would exist a $\bar{\tau }>\tau$ such that $\Phi \left(\bar{\tau }\right)=0,\dot{\Phi }\left(\bar{\tau }\right)<0$ which is impossible in $\Omega_{c}^{+}$. Similarly, only *XY*-arcs are possible in $\Omega_{c}^{-}$.     □

The structure implied by Theorem 14 is illustrated in Figure 4. Note that inside the sets $\Omega_{c}^{+},\Omega_{c}^{-}$, and $\bar{L}$, extremal lifts are precisely characterized. Furthermore, the results of section 4.1 (and particularly Equation 18) yield the characterization on the boundary *N*. What remains is then to determine the synthesis of these controls to the entire domain Ω_*c*_, as well as to determine the local optimality of the singular arc $\bar{L}$. The latter is addressed in the following section.

### 4.4. Optimality of Singular Arcs

We begin by proving that the singular arc is extremal, i.e. that it satisfies the necessary conditions presented in section 4.2 (note that it is interior by assumption). This is intuitively clear from Figure 4, since *X* and *Y* point to opposite sides along $\bar{L}$ by the definition of L.

**THEOREM 15**. *The line segment* $\bar{L}\subset L$ *is a singular arc*.

*Proof*. We find an expression for *u* = *u*(*x*) such that the vector *f*(*x*) + *u*(*x*)*g*(*x*) is tangent to $\bar{L}$ at *x*, i.e. we find the unique solution to

(55) $$\begin{array}{l} L_{f+ug}\left(\gamma \right)=0 \end{array}$$

Note that we can invert (50):

(56) $$\begin{array}{l} \begin{array}{l} f\left(x\right)=X\left(x\right)\\ g\left(x\right)=\frac{1}{M}\left(Y\left(x\right)-X\left(x\right)\right) \end{array} \end{array}$$

so that $f+ug=\left(1-\frac{u}{M}\right)X+\frac{u}{M}Y$. Thus,

$$\begin{array}{l} L_{f+ug}\left(\gamma \right)=\left(1-\frac{u}{M}\right)L_{X}\gamma +\frac{u}{M}L_{Y}\gamma \end{array}$$

Setting the above equal to zero, and solving for *u* = *u*(*x*) yields

(57) $$\begin{array}{l} u\left(x\right)=M\frac{L_{X}\gamma \left(x\right)}{L_{X}\gamma \left(x\right)-L_{Y}\gamma \left(x\right)} \end{array}$$

As *L*_*X*_γ < 0 and *L*_*Y*_γ > 0 on $\bar{\text{L}}$ by Proposition 11, we see that 0 < *u*(*x*) < *M*. We must also verify that the associated controlled trajectory (57) is extremal by constructing a corresponding lift. Suppose that *x*(*t*) solves

$$\begin{array}{l} \;\dot{x}=f\left(x\right)+u\left(x\right)g\left(x\right),\\ x\left(0\right)=q, \end{array}$$

for $q\in \bar{L}$. Let ϕ ∈ (R^2^)* such that

$$\begin{array}{l} \langle \phi ,g\left(q\right)\rangle =0,\;\langle \phi ,f\left(q\right)\rangle =1. \end{array}$$

Let λ(*t*) solve the corresponding adjoint Equation (24) with initial condition λ(0) = ϕ. Then the extremal lift Γ = ((*x, u*), λ) is singular if Φ(*t*) = 〈λ(*t*), *g*(*x*(*t*))〉 ≡ 0. By construction of *u*(*x*), the trajectory remains on $\bar{L}$ on some interval containing zero, and we can compute $\dot{\Phi }$ as [using (34)]

$$\begin{array}{l} \dot{\Phi }(t)=\langle \lambda (t),[f,g](x(t))\rangle \\ \;=\gamma (x(t))\langle \lambda (t),f(x(t)\rangle +\beta (x(t))\langle \lambda (t),g(x(t))\rangle \\ \;=\beta (x(t))\Phi (t), \end{array}$$

Note that we have used (43) and the fact that γ = 0 by our choice of *u*. Since Φ(0) = 0 by hypothesis, this implies that Φ(*t*) ≡ 0, as desired.     □

The above then verifies that $\bar{L}$ is a singular arc. Note that an explicit expression for *u* = *u*(*x*) was given in (52), which can be shown to be equivalent to (57).

Having shown that the singular arc $\bar{L}$ is extremal, we now investigate whether it is locally optimal for our time-optimization problem. The singular arc is of intrinsic order *k* if the first 2*k* − 1 derivatives of the switching function are independent of *u* and vanish identically on an interval *I*, while the 2kth derivative has a linear factor of *u*. We can compute [this is standard for control-affine systems (8)] that

(58) $$\begin{array}{l} \Phi^{2k}\left(t\right)=\langle \lambda \left(t\right),{\text{ad}}_{f}^{2k}\left(g\right)\left(x\left(t\right)\right)\rangle +u\left(t\right)\langle \lambda \left(t\right),\left[g,{\text{ad}}_{f}^{2k-1}\left(g\right)\right]\left(x\left(t\right)\right)\rangle , \end{array}$$

where ad_*Z*_ is the adjoint endomorphism for a fixed vector field *Z*:

(59) $$\begin{array}{l} {\text{ad}}_{Z}\left(V\right)=\left[Z,V\right], \end{array}$$

and powers of this operator are defined as composition. Fix an extremal lift Γ = ((*x, u*), λ) of a singular arc of order *k*. The Generalized Legendre-Clebsch condition (also known as the Kelley condition) (Ledzewicz and Schättler, 2012) states that a necessary condition for Γ to satisfy a minimization problem with corresponding Hamiltonian *H* is that

(60) $$\begin{array}{l} {\left(-1\right)}^{k}\frac{\partial }{\partial u}\frac{d^{2k}}{dt^{2k}}\frac{\partial H}{\partial u}\left(\lambda_{0},\lambda \left(t\right),x\left(t\right),u\left(t\right)\right)\geq 0 \end{array}$$

along the arc. Note that $\frac{\partial H}{\partial u}=\Phi$, so that the above is simply the *u* coefficient of the 2*k*-th time derivative of the switching function (multiplied by (−1)^*k*^). The order of the arc, as well as the Legendre-Clebsch condition, are addressed in Theorem 16.

**THEOREM 16**. *The singular control is of order one. Furthermore, for all times t such that* $x\left(t\right)\in \bar{L}$, 〈λ(*t*), [*g*, [*f, g*]](*x*(*t*))〉 > 0. *Thus, the Legendre-Clebsch condition is violated, and the singular arc* $\bar{L}$ *is not optimal*.

*Proof*. Along singular arcs we must have $\Phi \left(t\right),\dot{\Phi }\left(t\right),\ddot{\Phi }\left(t\right)\equiv 0$, and we can compute these derivatives using iterated Lie brackets as follows:

(61) $$\begin{array}{l} \begin{array}{l} \Phi \left(t\right)=\langle \lambda \left(t\right),g\left(x\left(t\right)\right)\rangle ,\\ \dot{\Phi }\left(t\right)=\langle \lambda \left(t\right),\left[f,g\right]\left(x\left(t\right)\right)\rangle ,\\ \ddot{\Phi }\left(t\right)=\langle \lambda \left(t\right),\left[f+ug,\left[f,g\right]\right]\left(x\left(t\right)\right)\rangle . \end{array} \end{array}$$

The final of the above in (61) can be simplified as

(62) $$\begin{array}{l} \ddot{\Phi }\left(t\right)=\langle \lambda \left(t\right),\left[f,\left[f,g\right]\right]\left(x\left(t\right)\right)\rangle +u\left(t\right)\langle \lambda \left(t\right),\left[g,\left[f,g\right]\right]\left(x\left(t\right)\right)\rangle \equiv 0, \end{array}$$

which is precisely (58) for *k* = 1. Order one is then equivalent to being able to solve this equation for *u*(*t*). Thus, 〈λ(*t*), [*g*, [*f, g*]](*x*(*t*))〉 > 0 will imply that the arc is singular of order one. We directly compute 〈λ(*t*), [*g*, [*f, g*]](*x*(*t*))〉 = 〈λ(*t*), [*g*, ad_*f*_(*g*)](*x*(*t*))〉. Using Equation (34) and recalling properties of the singular arc [γ = 0 and the remaining relations in (61), as well as basic “product rule” properties of the Lie bracket], we can show that

(63) $$\begin{array}{l} \left[g,\left[f,g\right]\right]=\left(L_{g}\gamma \right)f-\gamma \left[f,g\right]+\left(L_{g}\beta \right)g. \end{array}$$

Recall that for an extremal lift along the arc $\bar{L}$,

(64) $$\begin{array}{l} \begin{array}{l} \;\langle \lambda \left(t\right),g\left(x\left(t\right)\right)\rangle \equiv 0,\\ \langle \lambda \left(t\right),\left[f,g\right]\left(x\left(t\right)\right)\rangle \equiv 0\\ \;\langle \lambda \left(t\right),f\left(x\left(t\right)\right)\rangle \equiv 1. \end{array} \end{array}$$

The first two of the above follow from $\Phi ,\dot{\Phi }\equiv 0$, and the third is a consequence of *H* ≡ 0 [see (22)]. Equations (63) and (64) together imply that

(65) $$\begin{array}{l} \begin{array}{l} \langle \lambda \left(t\right),\left[g,\left[f,g\right]\right]\left(x\left(t\right)\right)\rangle =L_{g}\gamma \langle \lambda \left(t\right),f\left(x\left(t\right)\right)\rangle -\gamma \langle \lambda \left(t\right),\left[f,g\right]\left(x\left(t\right)\right)\rangle \\ \;+L_{g}\beta \langle \lambda \left(t\right),g\left(x\left(t\right)\right)\rangle \\ \;=L_{g}\gamma \left(x\left(t\right)\right)\\ \;=\frac{1}{M}\left(L_{Y}\gamma \left(x\left(t\right)\right)-L_{X}\gamma \left(x\left(t\right)\right)\right). \end{array} \end{array}$$

The last equality follows from the representation in (56). As *L*_*Y*_γ > 0 and *L*_*X*_γ < 0 along $\bar{\text{L}}$ (Proposition 11), 〈λ(*t*), [*g*, [*f, g*]](*x*(*t*))〉 > 0, as desired. Furthermore,

(66) $$\begin{array}{l} -\langle \lambda \left(t\right),\left[g,\left[f,g\right]\right]\left(x\left(t\right)\right)\rangle <0,\text{ or equivalently} \end{array}$$

(67) $$\begin{array}{l} {\left(-1\right)}^{1}\frac{\partial }{\partial u}\frac{d^{2}}{dt^{2}}\frac{\partial H}{\partial u}<0, \end{array}$$

showing that (60) is violated (substituting *k* = 1). Thus, $\bar{L}$ is not optimal.     □

Theorem 16 then implies that the singular arc is suboptimal, i.e. that $\bar{L}$ is “fast” with respect to the dynamics. In fact, we can compare times along trajectories using the “clock form,” a one-form on Ω. As one-forms correspond to linear functionals on the tangent space, and *f* and *g* are linearly independent, there exists a unique ω ∈ (*TΩ*)^*^ such that

(68) $$\begin{array}{l} \omega_{x}\left(f\left(x\right)\right)\equiv 1,\;\omega_{x}\left(g\left(x\right)\right)\equiv 0. \end{array}$$

In fact, we compute it explicitly:

(69) $$\begin{array}{l} \omega_{x}=\frac{g_{2}\left(x\right)dx^{1}-g_{1}\left(x\right)dx^{2}}{\det\left(f\left(x\right),g\left(x\right)\right)}. \end{array}$$

Then, along any controlled trajectory (*x, u*) defined on [*t*_0_, *t*_1_], the integral of ω computes the time *t*_1_ − *t*_0_:

(70) $$\begin{array}{l} \int_{x}\omega =\int_{t_{0}}^{t_{1}}\omega_{x(t)}(\dot{x}(t))\text{ d}t\\ \;=\int_{t_{0}}^{t_{1}}\omega_{x(t)}(f(x(t))+u(t)g(x(t))))\text{ d}t\\ \;=\int_{t_{0}}^{t_{1}}\omega_{x(t)}(f(x(t))\text{ d}t+\int_{t_{0}}^{t_{1}}u(t)\omega_{x(t)}(g(x(t))))\text{ d}t\\ \;=\int_{t_{0}}^{t_{1}}\;\text{d}t\\ \;=t_{1}-t_{0}. \end{array}$$

**Figure 5:**
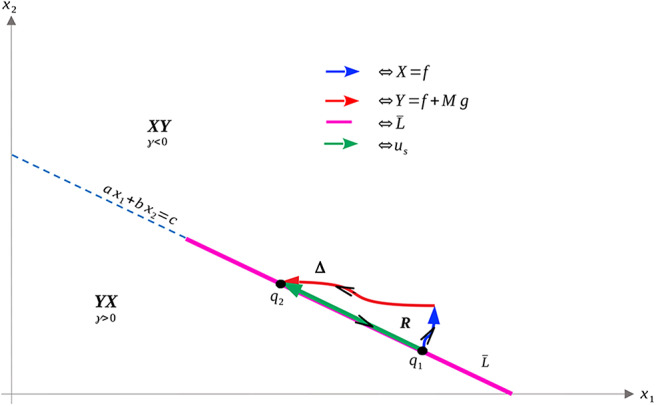
Both *XY* and singular trajectories taking *q*_1_ to *q*_2_.

We can then use ω and Stokes' Theorem to compare bang-bang trajectories with those on the singular arc. See Figure 5 below for a visualization of a singular trajectory connecting $q_{1},q_{2}\in \bar{L}$ and the corresponding unique *XY* trajectory connecting these points in $\Omega_{c}^{-}$ (note that uniqueness is guaranteed as long as *q*_1_ and *q*_2_ are sufficiently close).

Let *t*_*S*_ denote the time spent along the singular arc, *t*_*X*_ the time spent along the *X* arc, and *t*_*Y*_ the time spent along the *Y* arc. Denote by Δ the closed curve traversing the *X* and *Y* arcs positively and the singular arc negatively, with *R* as its interior. As *X* and *Y* are positively oriented (they have the same orientation as *f* and *g*), Stokes' Theorem yields

(71) $$\begin{array}{l} t_{X}+t_{Y}-t_{S}=\int_{\Delta }\omega =\int_{R}d\omega \end{array}$$

Taking the exterior derivative yields the two-form *dω* see Chapter 2 of (Ledzewicz and Schättler, 2012):

(72) $$\begin{array}{l} d\omega =-\frac{\gamma }{\det\left(f,g\right)}. \end{array}$$

As the determinant is everywhere positive (see the proof of Proposition 8), and *R* lies entirely in γ < 0, the integral on the right-hand side of (71) is positive, so that we have

(73) $$\begin{array}{l} t_{S}<t_{X}+t_{Y} \end{array}$$

**Figure 6:**
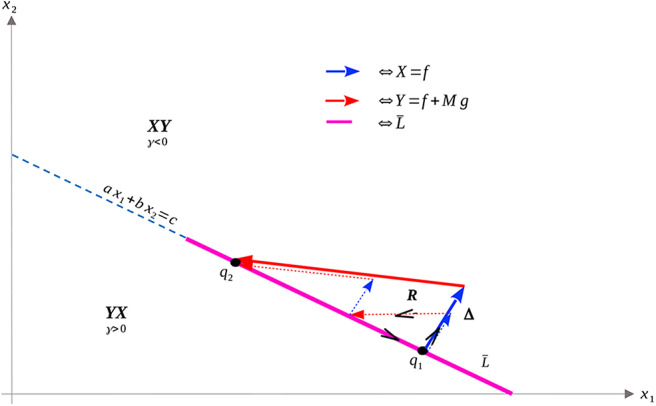
*XY* (solid) and *XYXY* (dashed) trajectories taking *q*_1_ to *q*_2_ in the region γ > 0. The time difference between the two trajectories can again be related to the surface integral in the region *R*, where γ < 0. The *XY* trajectory can then be seen to be slower in comparison.

Thus, time taken along the singular arc is shorter than that along the *XY* trajectory, implying that the singular arc is locally suboptimal for our problem (recall that we want to maximize time). Since local optimality is necessary for global optimality, trajectories should never remain on the singular arc for a measurable set of time points. This reaffirms the results of Theorem 16. A completely analogous statement holds for *YX* trajectories in the region γ > 0. We can also demonstrate, utilizing the same techniques, that increasing the number of switchings at the singular arc speeds up the trajectory (see Figure 6). This again reinforces Theorem 16, and implies that trajectories should avoid the singular arc to maximize the time spent in Ω_*c*_.

## 5. Characterization of Optimal Control

The results of sections 4.1, 4.2, 4.3, and 4.4 may now be combined to synthesize the optimal control introduced in section 3.

**THEOREM 17**. *For any α* ≥ 0, *the optimal control to maximize the time to reach a critical time is a concatenation of bang-bang and path-constraint controls. In fact, the general control structure takes the form*

(74) $$\begin{array}{l} {\left(YX\right)}^{n}u_{p}Y \end{array}$$

*where* (*YX*)^*n*^: = (*YX*)^*n*−1^*YX* *for* *n* ∈ N, *and the order should be interpreted as left to right. Here* *u*_*p*_ *is defined in* (*18*).

*Proof*. Formula (74) is simply a combination of the results presented previously. Note that singular arcs are never (locally) optimal, and hence do not appear in the equation. We also observe that *X* arcs are not admissible once the boundary *N* has been obtained, as an *X* arc always increases *V*. A *Y* arc may bring the trajectory back into int(Ω_*c*_), but a *YX* trajectory is no longer admissible, as the switching structure in $\Omega_{c}^{-}$ is *XY* (Theorem 14).

**Figure 7:**
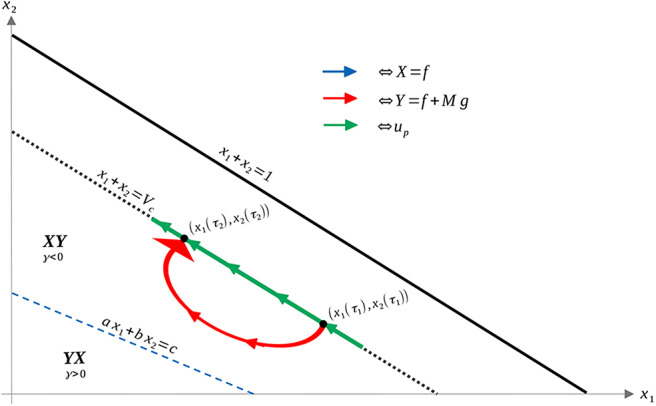
Comparison of *u*_*p*_*Yu*_*p*_ arc and an arc that remains on *N* (hence *u* ≡ *u*_*p*_) between the points [*S*(τ_1_), *R*(τ_1_)] and [*S*(τ_2_), *R*(τ_2_)], assuming that *u*_*p*_ remains feasible (that is, *u*_*p*_ ∈ [0, *M*]). Note that γ < 0 in the area of interest, and that a switching of a *Y* to an *X* arc is prohibited via the Maximum Principle. Thus, the only possibility is the curve illustrated, which leaves the boundary *N* for a *Y* arc before *u*_*p*_ becomes infeasible.

The only aspect that remains is to show that once *N* is reached, the only possible trajectories are either *u*_*p*_ given by (18) or *Y*, with at most one switching occurring between the two. That is, a local arc of the form *u*_*p*_*Yu*_*p*_ is either sub-optimal or non-feasible (equivalently, outside of the control set *U*). Suppose that such an arc is feasible, i.e., that for all such points in phase space, 0 ≤ *u*_*p*_ ≤ *M* [recall that *u*_*p*_ is defined via feedback in (18)]. Denote by τ_1_ and τ_2_ the times at which the switch onto and off of *Y* occurs, respectively. Since *u*_*p*_ decreases with *S*, feasibility implies that *u*_*p*_(*t*) ≤ *M* for all *t* ∈ [τ_1_, τ_2_]. Thus, we can consider the alternate feasible trajectory which remains on *N* between the points (*S*(τ_1_), *R*(τ_1_)) and (*S*(τ_2_), *R*(τ_2_)); see Figure 7 for an illustration. Call τ the time for such a trajectory. Then, using the clock-form ω and the positively-oriented curve Δ which follows *N* first and *Y* (in the reverse direction) second, we obtain similarly to (71),

(75) $$\begin{array}{l} \tau -\left(\tau_{2}-\tau_{1}\right)=-\int_{R}\frac{\gamma }{\det\left(f,g\right)}, \end{array}$$

where *R*: = int(Δ). Recalling that γ < 0 in *R* (see Figure 4), the previous equation implies that

(76) $$\begin{array}{l} \tau >\tau_{1}-\tau_{2}, \end{array}$$

i.e., a longer amount of time is achieved by remaining on the boundary *N*. Hence the arc *u*_*p*_*Yu*_*p*_ is sub-optimal if it is feasible, as desired.

The previous argument has one subtle aspect, as we used results from the Maximum Principle on the boundary set *N*, where technically it does not apply. However, the above still remains true, since we may approximate the boundary line *V* = *V*_*c*_ with a curve interior to Ω_*c*_ which remains feasible. By continuity, the time along such a curve can be made arbitrarily close to τ, and hence is still greater than τ_2_ − τ_1_, implying that *u*_*p*_*Yu*_*p*_ is sub-optimal.     □

Note that in Theorem 17, the switchings must occur across the singular arc $\bar{L}$, if it exists (recall that it is not admissible if α = 0). The control *u*_*p*_ is determined along the boundary of Ω_*c*_, and provides the synthesis between interior and boundary controls.

We finally include a technical result, which eliminates the optimality of the constrained (boundary) control *u*_*p*_ in certain cases.

**Proposition 18**. *Assume that the maximal dose M is as in Proposition 2*:

(77) $$\begin{array}{l} M>\frac{\left(1-V_{c}\right)\left(1-p_{r}\right)}{d} \end{array}$$

*If the optimal control becomes maximal in* $\Omega_{c}^{-}$ (*i.e., u* = *M in this region*), *then the control cannot take the boundary value u*_*p*_ (*Equation 18*) *on an interval. Equivalently, an optimal control cannot end in the form Yu*_*p*_.

*Proof*. Note that if u_*_ = *Y* and reaches *N* at the point *x*, then the Lie derivative *L*_*Y*_*V*(*x*) must satisfy

(78) $$\begin{array}{l} L_{Y}V\left(x\right)\geq 0 \end{array}$$

as *V* must be increasing along the *Y* vector field, since it reaches *N*. But by Proposition 2, this implies that

$$\begin{array}{l} x_{1}\leq x_{1}^{*} \end{array}$$

Proposition 3 then implies that *u*_*p*_ is unfeasible in this region, completing the proof.     □

## 6. Numerical Results

In this section, we provide numerical examples of the analytical results obtained in previous sections. All figures in this section were obtained using the GPOPS-II MATLAB software (Patterson and Rao, 2014). Parameters and initial values are given in Table 1 shown below, unless stated otherwise.

**Table 1:** Parameter values and initial conditions used throughout section 6, unless stated otherwise.

**Parameters**	**Interpretation**	**Value**
$x_{1}^{0}$	Initial sensitive population	10^−2^
$x_{2}^{0}$	Initial resistant population	0
α	Induced resistance rate due to the presence of the drug	10^−2^
*d*	Drug cytotoxicity parameter	1
ϵ	Drug-independent resistance rate	10^−6^
*p*_*r*_	Resistant growth fraction	0.2
*t*_0_	Initial time	0
*M*	Maximum drug dosage	5
*V*_*c*_	Tumor volume defining treatment failure	0.9

**Figure 8:**
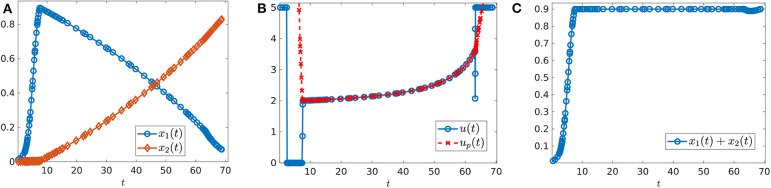
Numerical solution of the optimal control problem with *d* = 0.05, α = 0.005, and the remainder of parameters as in Table 1. **(A)** Sensitive (*x*_1_) and resistant (*x*_2_) temporal dynamics. **(B)** Control structure of form *YXu*_*p*_*Y*. **(C)** Volume dynamics. Note that the trajectory remains on the line *V* = *V*_*c*_ for most times, with corresponding control *u* = *u*_*p*_.

**Figure 9:**
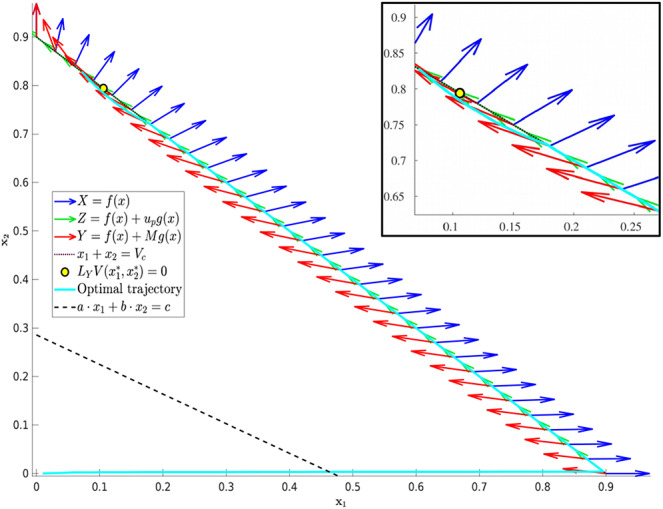
Phase plane corresponding to Figure 8. Trajectory which optimal control is of the form *YXu*_*p*_*Y* with parameter values as in Table 1 except for α = 0.005 and *d* = 0.05. The yellow dot in the figure represents the $\left(x_{1}^{*},x_{2}^{*}\right)$ point at which *Y*(*x*) is tangent to the sliding surface. Here, $\left(x_{1}^{*},x_{2}^{*}\right)=\left(0.1059,0.7941\right)$. As proven in Proposition 2, for points on the line *N*, the tumor volume will decrease along the *Y*(*x*) direction if *x*_1_ > 0.1059 and will increase for *x*_1_ < 0.1059.

**Figure 10:**
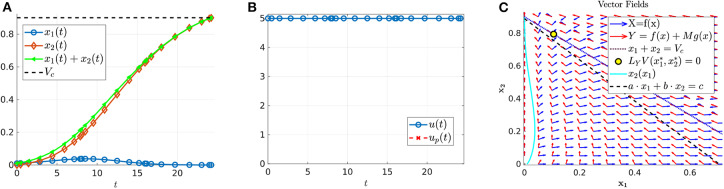
Numerical solution of optimal control problem with *d* = 0.05, α = 0.1, and the remainder of parameters as in Table 1. **(A)** Sensitive (*x*_1_), resistant (*x*_2_), and volume (*x*_1_ + *x*_2_) temporal dynamics. **(B)** Control structure of form *Y*, i.e., an entirely upper corner control. **(C)** Phase plane dynamics, plotted with relevant vector fields.

Theorem 17 characterizes the qualitative form of the optimal control:

(79) $$\begin{array}{l} u_{*}={\left(YX\right)}^{n}u_{p}Y, \end{array}$$

where *n* is the number of interior switches, *u*_*p*_ the sliding control (18), and *X* and *Y* denote the lower and upper corner controls *u* = 0 and *u* = *M*, respectively. We begin by computing sample controls (see Figures 8, 10). Note that the optimal control in Figure 8B takes the form *YXu*_*p*_*Y*, while that of Figure 10B is an upper corner control *Y*. The phase plane dynamics corresponding to Figure 8 are also provided in Figure 9. In both cases the cytotoxic parameter was fixed at *d* = 0.05, while the induced rate of resistance α varies between α = 0.005 in Figure 8 and α = 0.1 in Figure 10. Note that for the smaller value of α (Figure 8), a longer period of treatment success is observed, as the time to treatment failure is approximately 70 time units; compare this with *t*_*c*_ = 24.2 in Figure 10. This result is intuitive, as the treatment less likely to induce resistance is able to be more effective when optimally applied.

**Figure 11:**
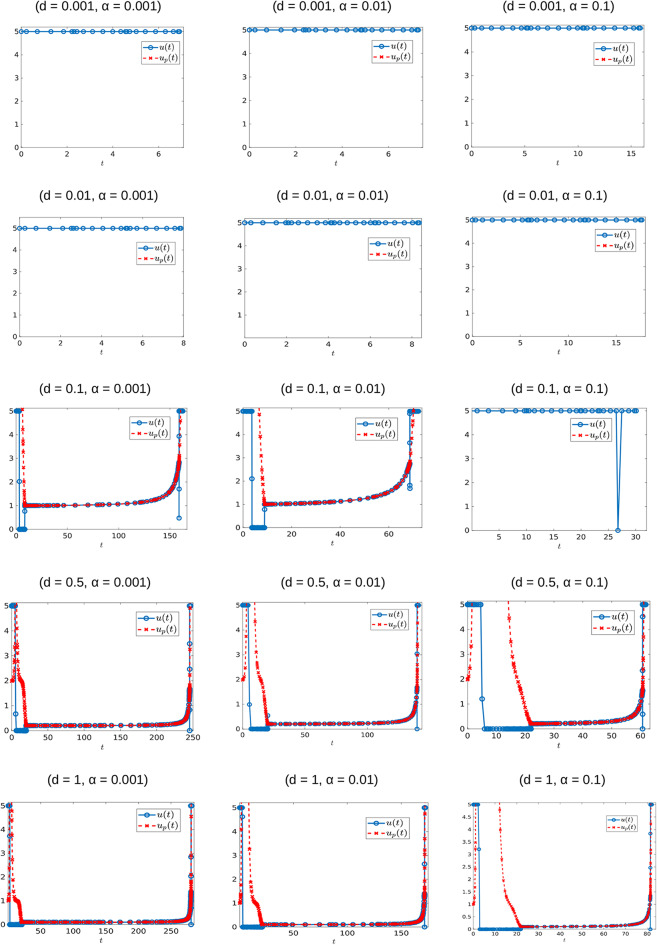
Optimal control structures for different α and *d* values. The blue curve is the computed optimal control, while the red curve is the feedback control along on the boundary of *N*, which may or may not be optimal or even feasible.

**Figure 12:**
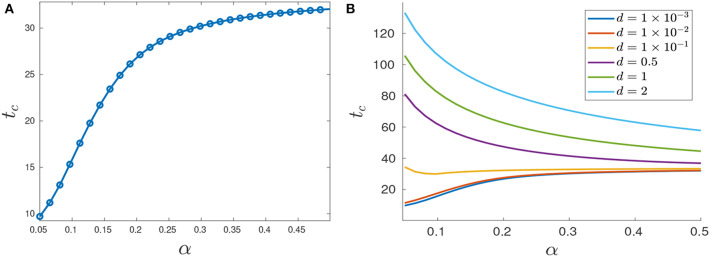
Variation in *t*_*c*_ as a function of α. **(A)** Treatment success time *t*_*c*_ for *d* = 0.001 with varying α values. **(B)** Functional dependence of *t*_*c*_ on α for different *d* parameters. Note that for small *d*, *t*_*c*_ increases as a function of α, but that this trend is reversed if *d* is further increased.

The generality of the previous statement is investigated in Table 2 and Figures 11, 12. The computed optimal times *t*_*c*_ suggest that when the cytotoxicity of the drug (*d*) is small, higher induction rates (α) actually increase treatment efficacy. For example, for *d* = 0.001 treatment response increases as α increases (Figure 12A). This could be explained from the fact that sensitive cells have a higher growth rate than resistant cells (assumption *p*_*r*_ < 1). Thus, when the chemotherapeutic drug has a low effectiveness (small *d*) a larger α value actually helps to reduce the sensitive population size, and therefore extends the time *t*_*c*_ at which the tumor volume exceeds its critical value *V*_*c*_.

**Table 2:** Optimal time *t*_*c*_ for each of the computed controls appearing in Figure 11.

**d**	**α = 0.001**	**α = 0.01**	**α = 0.1**
0.001	6.91	7.28	15.83
0.01	7.87	8.34	17.66
0.1	162.53	72.14	30.09
0.5	246.56	140.93	61.81
1	281.25	172.26	82.13

The situation is reversed when we consider larger values of *d* because in this case it would take more time for the tumor to grow to its critical volume *V*_*c*_ if the drug effectiveness is large enough; see for example the row *d* = 0.5 in Table 2, and the corresponding purple curve in Figure 12B. Figure 12B provides the critical time as a function of α for multiple cytotoxicities *d*; note the qualitative change in *t*_*c*_ as *d* increases.

Examining Figure 11 and Table 2 also suggests that as *d* increases, the feedback control *u*_*p*_ becomes optimal on an interval [*t*_1_, *t*_2_] with 0 < *t*_1_ < *t*_2_ < *t*_*c*_. More numerical results are provided in section 7.3.

## 7. Additional Results

### 7.1. Structural Identifiability

For completeness, we discuss the identifiability of system (1). As our focus in this work has been on control structures based on the presence of drug-induced resistance, we rely on the ability to determine whether, and to what degree, the specific chemotherapeutic treatment is generating resistance.

Ideally, we envision a clinical scenario in which cancer cells from a patient are cultured in an *ex vivo* assay (for example, see Silva et al., 2017) prior to treatment. Parameter values are then calculated from treatment response dynamics in the assay, and an optimal therapy regime is implemented based on the theoretical work described below. Thus, identifying patient-specific model parameters, specially the induced-resistance rate α, is a necessary step in determining the control structures to apply. In this section, we address this issue, and prove that all parameters are structurally identifiable, as well as demonstrate a specific set of controls that may be utilized to determine α. A self-contained discussion is presented; for more details on theoretical aspects, see Sontag (2017) and the references therein. Other recent works related to identifiability in the biological sciences (as well as *practical identifiability*) can be found in Eisenberg and Jain (2017) and Villaverde et al. (2016).

We first formulate our dynamical system, and specify the input and output variables. The treatment *u*(*t*) is the sole input. Furthermore, we assume that the only clinically observable output is the net tumor volume *V*(*t*):

(80) $$\begin{array}{l} V\left(t\right):=x_{1}\left(t\right)+x_{2}\left(t\right). \end{array}$$

That is, we do not assume real-time measurements of the individual sensitive and resistant sub-populations. We note that in some instances, such measurements may be possible; however for a general chemotherapy, the precise resistance mechanism may be unknown *a priori*, and hence no biomarker with the ability to differentiate cell types may be available.

Treatment is initiated at time *t* = 0, at which we assume an entirely sensitive population:

(81) $$\begin{array}{l} x_{1}\left(0\right)=x_{1}^{0},\;x_{2}\left(0\right)=0. \end{array}$$

Here $0<x_{1}^{0}<1$, so that (*x*_1_(*t*), *x*_2_(*t*)) ∈ Ω for all *t* ≥ 0. We note that *x*_2_(0) = 0 is not restrictive, and similar results may derived under the more general assumption 0 ≤ *x*_2_(0) < 1. The condition *x*_2_(0) = 0 is utilized both for computational simplicity and since *x*_2_(0) is generally small (assuming a non-zero detection time, and small drug-independent resistance parameter ϵ; see Greene et al., 2019 for a discussion).

As formulated in section 7.2.1, the above then allows us to formulate our system (1) in input/output form, where the input *u*(*t*) appears affinely:

(82) $$\begin{array}{l} \begin{array}{l} \dot{x}\left(t\right)=f\left(x\left(t\right)\right)+u\left(t\right)g\left(x\left(t\right)\right),\\ x\left(0\right)=x_{0}, \end{array} \end{array}$$

where (as defined on Equations (9) and (10)) *f* and *g* are

(83) $$\begin{array}{l} f\left(x\right)=\left(\begin{array}{c} \left(1-\left(x_{1}+x_{2}\right)\right)x_{1}-\epsilon x_{1}\\ p_{r}\left(1-\left(x_{1}+x_{2}\right)\right)x_{2}+\epsilon x_{1} \end{array}\right), \end{array}$$

(84) $$\begin{array}{l} g\left(x\right)=\left(\begin{array}{c} -\left(\alpha +d\right)\\ \alpha \end{array}\right)x_{1}, \end{array}$$

and *x*(*t*) = (*x*_1_(*t*), *x*_2_(*t*)). As is standard in control theory, the output is denoted by the variable *y*, which in this work corresponds to the total tumor volume:

(85) $$\begin{array}{l} \begin{array}{l} y\left(t,p\right):=h\left(x\left(t\right),u\left(t\right),p\right)\\ \;=x_{1}\left(t\right)+x_{2}\left(t\right). \end{array} \end{array}$$

Note that *x*_1_(*t*), *x*_2_(*t*) depend on both the input *u*(*t*) and parameters *p*. A system in form (82) is said to be *uniquely structurally identifiable* if the map (*u*(*t*), *p*) ↦ (*u*(*t*), *y*(*t, p*)) is injective almost everywhere (Meshkat and Seth, 2014; Eisenberg and Jain, 2017), where *p* is the vector of parameters to be identified. In this work,

(86) $$\begin{array}{l} p=\left(x_{1}^{0},d,\alpha ,\epsilon ,p_{r}\right). \end{array}$$

*Local identifiability* and *non-identifiability* correspond to the map being finite-to-one and infinite-to-one, respectively. Our objective is then to demonstrate unique structural identifiability for model system (82) [or equivalently (1)], and hence recover all parameter values *p* from only measurements of the tumor volume *y*. We also note that the notion of identifiability is closely related to that of *observability*; for details Anguelova (2004); Sontag (1979) are good references.

To analyze identifiability, we utilize results appearing in, for example (Hermann and Krener, 1977; Wang and Sontag, 1989; Sontag and Wang, 1991), and hence frame the issue from a differential-geometric perspective. Our hypothesis is that perfect (hence noise-free) input-output data is available and in particular, for differentiable controls, that we can compute *y* and its derivatives. We thus, for example, make measurements of

(87) $$\begin{array}{l} \begin{array}{l} y\left(0\right)=h\left(x\left(0\right)\right),\\ \dot{y}\left(0\right)=\frac{d}{dt}{|}_{t=0}h\left(x\left(t\right)\right) \end{array} \end{array}$$

for appropriately chosen inputs, and relate their values to the unknown parameter values *p*. If there exist inputs *u*(*t*) such that the above system of equations may be solved for *p*, the system is identifiable. The right-hand sides of (87) may be computed in terms of the Lie derivatives of the vector fields *f* and *g* in system (82). We recall the definition of Lie differentiation *L*_*X*_*H* of a *C*^ω^ function *H* by a *C*^ω^ (i.e. real-analytic) vector field *X*:

(88) $$\begin{array}{l} L_{X}H\left(x\right):=\nabla H\left(x\right)\cdot X\left(x\right). \end{array}$$

Here the domain of both *X* and *H* is the first-quadrant triangular region Ω, seen as a subset of the plane, and the vector fields and output function are *C*^ω^ on an open set containing Ω (in fact, they are given by polynomials, so they extend as analytic functions to the entire plane). Iterated Lie derivatives are well-defined, and should be interpreted as function composition, so that for example *L*_*Y*_*L*_*X*_*H* = *L*_*Y*_(*L*_*X*_*H*), and $L_{X}^{2}H=L_{X}\left(L_{X}H\right)$.

More formally, let us introduce the observable quantities corresponding to the zero-time derivatives of the output *y* = *h*(*x*),

(89) $$\begin{array}{l} Y\left(x_{0},U\right)=\frac{d^{k}}{dt^{k}}{|}_{t=0}h\left(x\left(t\right)\right), \end{array}$$

where *U* ∈ *R*^*k*^ is the value of the control *u*(*t*) (without loss of generality, a polynomial of degree *k* − 1) and its derivatives evaluated at *t* = 0: *U* = (*u*(0), *u*′(0), ..., *u*^(*k*−1)^(0)). Here *k* ≥ 0, indicating that the *k*^th^-order derivative *Y* may expressed as a polynomial in the components of *U* (Sontag and Wang, 1991). The initial conditions *x*_0_ appear due to evaluation at *t* = 0. The observation space is then defined as the span of the elements *Y*(*x*_0_, *U*):

(90) $$\begin{array}{l} F_{1}:={\text{span}}_{\text{R}}\left\{Y\left(x_{0},U\right)\;|\;U\in {\text{R}}^{k},k\geq 0\right\}. \end{array}$$

Conversely, we also define span of iterated Lie derivatives with respect to the output *h* and vector fields *f*(*x*) and *g*(*x*):

(91) $$\begin{array}{l} F_{2}:={\text{span}}_{\text{R}}\left\{L_{i_{1}}\ldots L_{i_{k}}h\left(x_{0}\right)\;|\;\left(i_{1},\ldots i_{k}\right)\in {\left\{g,f\right\}}^{k},k\geq 0\right\}. \end{array}$$

Wang and Sontag (1989) proved that *F*_1_ = *F*_2_, so that the set of “elementary observables” may be considered as the set of all iterated Lie derivatives *F*_2_. Hence, identifiability may be formulated in terms of the reconstruction of parameters *p* from elements in *F*_2_. Parameters *p* are then identifiable if the map

(92) $$p\mapsto \left(L_{i_{1}}\ldots L_{i_{k}}h(x_{0})\;|\;(i_{1},\ldots i_{k})\in {\left\{g,f\right\}}^{k},k\geq 0\right)$$

is one-to-one. For the remainder of this section, we investigate the mapping defined in (92).

Computing the Lie derivatives and recalling that *x*_0_ = (*S*_0_, 0) we can recursively determine the parameters *p*:

(93) $$\begin{array}{l} \begin{array}{l} x_{1}^{0}=h\left(x_{0}\right),\\ d=-\frac{L_{g}h\left(x_{0}\right)}{x_{1}^{0}},\\ \alpha =\frac{L_{g}^{2}h\left(x_{0}\right)}{dx_{1}^{0}}-d,\\ \epsilon =\frac{L_{f}L_{g}h\left(x_{0}\right)}{dx_{1}^{0}}+1-x_{1}^{0},\\ p_{r}=\frac{x_{1}^{0}}{1-x_{1}^{0}}+\frac{L_{g}L_{f}h\left(x_{0}\right)}{\alpha x_{1}^{0}\left(1-x_{1}^{0}\right)}-\left(1+\frac{d}{\alpha }\right)\left(1-\frac{x_{1}^{0}}{1-x_{1}^{0}}\right). \end{array} \end{array}$$

Since *F*_1_ = *F*_2_, all of the above Lie derivatives are observable via appropriate treatment protocols. For an explicit set of controls and corresponding relations to measurable quantities [elements of the form (89)], see Greene et al. (2019). Thus, we conclude that all parameters in system (1) are identifiable, which allows us to investigate optimal therapies dependent upon *a priori* knowledge of the drug-induced resistance rate α.

### 7.2. Existence Results

For the problem presented in section 3, we are going to verify that the supremum of times *t*_*c*_(*u*) for u∈U [with *t*_*c*_(*u*) as defined in Equation (6)] is obtained by some $u_{*}\in U$, i.e., that an optimal control exists. This involves two distinct steps: (1) proving that the supremum is finite, and (2) that it is obtained by at least one admissible control. The following two subsections verify these claims.

#### 7.2.1. Finiteness of the Supremum

We prove that

(94) $$\begin{array}{l} \underset{u\in U}{\sup}t_{c}\left(u\right)<\infty \end{array}$$

for the control system introduced in section 3. The result depends crucially on (3), and the fact that the globally asymptotically stable state (0, 1) is disjoint from the dynamic constraint *x* ∈ Ω_*c*_ (see Equation (13)). That is, *V*_*c*_ < 1 is necessary for the following subsequent result to hold, and generally an optimal control will not exist if *V*_*c*_ = 1 or if the path constraint (13) is removed.

Our control system has the form

(95) $$\begin{array}{l} \dot{x}=f\left(x\right)+u\left(t\right)g\left(x\right), \end{array}$$

where *x* ∈ Ω, u∈U, and the vector fields *f, g*:Ω → R^2^ are continuously differentiable. Note that the above vector field is affine (and thus continuous) in the control *u*. Fix the initial condition

(96) $$\begin{array}{l} x\left(0\right)=x_{0}, \end{array}$$

with *x*_0_ ∈ Ω. Recall that all solutions of (95) and (96) approach the fixed point $\bar{x}:=\left(0,1\right)\in \Omega$. That is, for all u∈U,

(97) $$x_{u}(t)\;\overset{t\to \infty }{\to }\;\bar{x}.$$

Note that we explicitly denote the dependence of the trajectory on the control *u*, and the above point $\bar{x}$ is independent of the control *u*.

For any compact subset *E* of Ω such that $x_{0}\in E,\bar{x}\notin E$, we associate to each control (and hence to its corresponding trajectory) a time *t*_*E*_(*u*) such that

(98) $$\begin{array}{l} t_{E}\left(u\right)=\max\left\{T\;|\;x_{u}\left(t\right)\in E\text{ for all }t\leq T\right\}. \end{array}$$

The above is well-defined (as a maximum) for each control *u*, since by assumption *x*_0_ ∈ *E* and each trajectory asymptotically approaches $\bar{x}\notin E$, *x*_*u*_ is continuous, and *E* is compact.

**THEOREM 19**. *Define*

(99) $$\begin{array}{l} T_{*}=\underset{u\in U}{\sup}t_{E}\left(u\right). \end{array}$$

With the above construction, T_*_ *is finite*.

*Proof*. Consider the sets *K, V* ⊂ R^2^, with *V* being an open neighborhood of the steady state $\bar{x}=\left(0,1\right)$ and *K* a compact set in R^2^ such that

$$\begin{array}{c} (0,1)\in V⊊K\text{ and }K\cap \{(x_{1},x_{2})\in {\text{R}}^{2}:x_{1},x_{2}\geq 0\\ \text{and }0\leq x_{1}+x_{2}\leq V_{c}\}=\emptyset . \end{array}$$

By contradiction, suppose that T_*_ is not finite, so we can find a sequence of controls ${\left\{v_{k}\right\}}_{k=1}^{\infty }$ in U satisfying

(100) $$d_{\infty }\left(x(t,v_{k}),K\right)\geq \epsilon \text{ for all }t\leq t_{k},\text{with }t_{k}\to \infty .$$

where *d*_∞_ denotes the supremum metric and, for each *k* ∈ N, *x*(*t, v*_*k*_) is the solution of the IVP:

(101) $$\begin{array}{l} \begin{array}{l} \dot{x}=f\left(x\right)+v_{k}\left(t\right)g\left(x\right),\\ x\left(0\right)=x_{0}, \end{array} \end{array}$$

Our aim is to find a control u∈U such that *x*(*t, u*), solution of system (101), does not enter *K* for any *t* > 0. Recall that by the Banach-Alaoglu theorem, the ball

(102) $$B(L^{\infty }([0,\infty )))=\{u\in L^{\infty }([0,\infty )):\|u{\|}_{\infty }\leq M\}$$

is a compact set on the weak^*^ topology and metrizable. Thus, the sequence ${\left\{v_{k}\right\}}_{k=1}^{\infty }$ must have a weak^*^−convergent subsequence ${\left\{u_{j}\right\}}_{j=1}^{\infty }$ which converges to a control *u* ∈ *L*^∞^([0, ∞)). In other words, for every ψ ∈ *L*^1^([0, ∞))

(103) $$\begin{array}{l} \underset{j\to \infty }{\lim}\int_{\left[0,\infty \right)}\psi u_{j}d\mu =\int_{\left[0,\infty \right)}\psi ud\mu , \end{array}$$

where μ is the usual Lebesgue measure. This means that the sequence ${\left\{u_{j}\right\}}_{j=1}^{\infty }$ converges to *u* with respect to the weak^*^ topology on *L*^∞^([0, ∞)) as the dual of *L*^1^([0, ∞)).

We next prove that $\lim_{j\to \infty }||x\left(t,u\right)-x\left(t,u_{j}\right){||}_{\infty }=0$ for all *t* ∈ [*t*_*k*−1_, *t*_*k*_] and all *k* ∈ N. In order to do so define

$$x_{k-1}=x_{0}+\int_{0}^{t_{k-1}}\left[f\left(x\left(s\right)\right)+u\left(s\right)g\left(x\left(s\right)\right)\right]ds$$

for any *t*_*k*−1_ ∈ [0, ∞), where *x* solves the IVP

(104) $$\begin{array}{l} \begin{array}{l} \dot{x}=f\left(x\right)+v_{k}\left(t\right)g\left(x\right),\\ x\left(t_{k-1}\right)=x_{k-1}. \end{array} \end{array}$$

Notice that the fact that the equilibrium (0, 1) is globally asymptotically stable on $\left\{\left(x_{1},x_{2}\right)\in {\text{R}}^{2}:x_{1},x_{2}\geq 0\;\text{and}\;0<x_{1}+x_{2}\leq V_{c}\right\}$ implies that *x*_*k*−1_ is well-defined for any *k* ∈ N.

The integral form of (104) is given by

(105) $$\begin{array}{l} F\left(t,x,v_{k}\right)=x_{k-1}+\int_{t_{k-1}}^{t}\left[f\left(x\right)+v_{k}\left(s\right)g\left(x\right)\right]ds. \end{array}$$

With the help of the *t*_*k*_'s from (100) and assuming (without loss of generality) that *t*_*k*_ increases as *k* goes to infinity, we write the set [0, ∞) as the countable union of finite closed intervals:

$$\left[0,\infty \right)=\underset{k\in \text{N}}{⋃}\left[t_{k-1},t_{k}\right]\text{ where }t_{0}=0.$$

Let *w*_*j, k*_ and *w* denote the functions *u*_*j*_ and *u* restricted to the interval [*t*_*k*−1_, *t*_*k*_], respectively. Thus, the sequence ${\left\{w_{j,k}\right\}}_{j=1}^{\infty }$ converges weakly* to *w* on [*t*_*k*−1_, *t*_*k*_]:

(106) $$\begin{array}{l} \underset{j\to \infty }{\lim}||x\left(t,w\right)-x\left(t,w_{j,k}\right){||}_{\infty }\\ =\underset{j\to \infty }{\lim}||F\left(t,x,w\right)-F\left(t,x,w_{j,k}\right){||}_{\infty } \end{array}$$

(107) $$=\underset{j\to \infty }{\lim}{\left\|\int_{t_{k-1}}^{t}w(s)g(x)ds-\int_{t_{k-1}}^{t}w_{j,k}(s)g(x)ds\right\|}_{\infty }$$

(108) $$\begin{array}{l} =\underset{j\to \infty }{\lim}{\left\|\int_{t_{k-1}}^{t}[w_{j,k}(s)-w(s)]g(x)ds\right\|}_{\infty } \end{array}$$

(109) $$\begin{array}{l} =0\text{ for all }t\in \left[t_{k-1},t_{k}\right]\text{.} \end{array}$$

Since this result is independent of *k*, this implies that

(110) $$\begin{array}{l} d_{\infty }(x(t,u),K)=\underset{j\to \infty }{\lim}d_{\infty }(x(t,u_{j}),K)\geq \epsilon \text{ for all}\\ \;t\in [t_{k-1},t_{k}],\text{independently of k}\in \text{N}. \end{array}$$

The corresponding trajectory *x*(*t, u*) thus never enters *K*, contradicting the the global stability of $\bar{x}$. Hence, T_*_ must be finite, as desired.     □

For the system and control problem defined in sections 2 and 3, the above theorem implies that $\sup_{u\in U}t_{c}\left(u\right)$ is finite by taking *E* = Ω_*c*_.

#### 7.2.2. Supremum as a Maximum

Here we provide a general proof for the existence of optimal controls for systems of the form (95), assuming the set of maximal times is bounded above, which we have proven for our system in section 7.2.1. For convenience, we make the proof as self-contained as possible (one well-known result of Filippov will be cited), and state the results in generality which we later apply to the model of induced resistance. Arguments are adapted primarily from the textbook of Bressan and Piccoli (2007).

Consider again general control systems as in section 7.2.1. Solutions (or trajectories) of (95) will be defined as absolutely continuous functions for which a control u∈U exists such that (*x*(*t*), *u*(*t*)) satisfy (95) a.e., in their (common) domain [*a, b*].

It is easier and classical to formulate existence with respect to differential inclusions. That is, define the multi-function

(111) $$\begin{array}{l} F\left(x\right)=\left\{f\left(x\right)+\omega g\left(x\right)\;|\;\omega \in U\right\}. \end{array}$$

Thus, the control system (95) is clearly related to the inclusion

(112) $$\begin{array}{l} \dot{x}\in F\left(x\right). \end{array}$$

The following theorem (see Filippov, 1967 for a proof) makes this relationship precise.

**THEOREM 20**. *An absolutely continuous function* *x*:[*a, b*] ↦ R^2^ *is a solution of (95) if and only if it satisfies (112) almost everywhere*.

We first prove a lemma demonstrating that the set of trajectories is closed with respect to the sup-norm ||·||_∞_ if all the sets of velocities *F*(*x*) are convex.

LEMMA 21. *Let* *x*_*k*_ *be a sequence of solutions of (95) converging to* *x* *uniformly on* [*0*, *T*]. *If the graph of* (*t, x*(*t*)) *is entirely contained in Ω, and all the sets* *F*(*x*) *are convex, then* *x* *is also a solution of (95)*.

*Proof*. By the assumptions on *f, g*, the sets *F*(*x*) are uniformly bounded as (*t, x*) range in a compact domain, so that *x*_*k*_ are uniformly Lipschitz, and hence *x* is Lipschitz as the uniform limit. Thus *x* is differentiable a.e., and by Theorem 20, it is enough to show that

(113) $$\begin{array}{l} \dot{x}\left(t\right)\in F\left(x\left(t\right)\right) \end{array}$$

for all *t* such that the derivative exists.

Assume not, i.e., that the derivative exists at some τ, but *ẋ*(τ)∉*F*(*x*(τ)). Since *F*(*x*(τ)) is compact and convex, and *ẋ*(τ) is closed, the Hyperplane Separation Theorem implies that there exists a hyperplane separating *F*(*x*(τ)) and *ẋ*(τ). That is, there exists an ϵ > 0 and a (without loss of generality) unit-vector *p* ∈ R^2^ such that

(114) $$\begin{array}{l} \langle p,y\rangle \leq \langle p,\dot{x}\left(\tau \right)\rangle -3\epsilon , \end{array}$$

for all *y* ∈ *F*(*x*(τ)). By continuity, there exists δ > 0 such that for |*x*′ − *x*(τ)| ≤ δ

(115) $$\begin{array}{l} \langle p,y\rangle \leq \langle p,\dot{x}\left(\tau \right)\rangle -2\epsilon , \end{array}$$

for all *y* ∈ *F*(*x*′). Since *x* is differentiable at τ, we can choose τ′ > τ such that

(116) $$\begin{array}{l} \begin{array}{l} |\frac{x\left(\tau^{\prime }\right)-x\left(\tau \right)}{\tau^{\prime }-\tau }-\dot{x}\left(\tau \right)|<\epsilon ,\\ \;|x\left(t\right)-x\left(\tau \right)|<\delta , \end{array} \end{array}$$

for all *t* ∈ [τ, τ′]. Equation (116) and uniform convergence then implies that, as *p* is a unit vector,

(117) $$\langle p,\frac{x_{k}(\tau^{\prime })-x_{k}(\tau )}{\tau^{\prime }-\tau }\rangle \overset{k\to \infty }{\to }\langle p,\frac{x(\tau^{\prime })-x(\tau )}{\tau^{\prime }-\tau }\rangle \geq \langle p,\dot{x}(\tau )\rangle -\epsilon .$$

On the other hand, since *ẋ*(*t*) ∈ *F*(*x*′) for *t* ∈ [τ, τ′], Equation (115) implies that for *k* sufficiently large,

(118) $$\begin{array}{l} \langle p,\frac{x_{k}\left(\tau^{\prime }\right)-x_{k}\left(\tau \right)}{\tau^{\prime }-\tau }\rangle =\frac{1}{\tau^{\prime }-\tau }\int_{\tau }^{\tau^{\prime }}\langle p,\dot{x}\left(t\right)\rangle \text{d}t\leq \langle p,\dot{x}\left(\tau \right)\rangle -2\epsilon . \end{array}$$

Clearly, (117) and (118) contradict one another, so that (113) must be true, as desired.     □

We now restate the optimal control problem associated to (95). Let *S* denotes the set of admissible terminal conditions, *S* ⊂ R × R^2^, and ϕ:R × R^2^ ↦ R a cost function. We would like to maximize ϕ(*T, x*(*T*)) over admissible controls with initial and terminal constraints:

(119) $$\begin{array}{l} \begin{array}{c} \underset{u\in U,T\geq 0}{\max}\phi \left(T,x\left(T,u\right)\right),\\ x\left(0\right)=x_{0},\left(T,x\left(T\right)\right)\in S. \end{array} \end{array}$$

We now state sufficient conditions for such an optimal control to exist.

**THEOREM 22**. *Consider the control system (95) and corresponding optimal control problem (119). Assume the following*:

1. *The objective ϕ is continuous*.
2. *The sets of velocities F*(*x*) *are convex*.
3. *The trajectories x remain uniformly bounded*.
4. *The target set S is closed*.
5. *A trajectory satisfying the constraints in (119) exists*.
6. *S is contained in some strip* [*0*, *T*] × R^2^, *i.e. the set of final times (for free-endpoint problems) can be uniformly bounded*.

*If the above items are all satisfied, an optimal control exists*.

*Proof*. By assumption, there is at least one admissible trajectory reaching the target set *S*. Thus, we can construct a sequence of controls *u*_*k*_:[0, *T*_*k*_] ↦ *U* whose corresponding trajectories *x*_*k*_ satisfy

(120) $$\begin{array}{l} \;x_{k}(0)=x_{0},\\ (T_{k},x_{k}(T_{k}))\in S,\\ \phi (T_{k},x(T_{k}))\overset{k\to \infty }{\to }\underset{u\in U,\bar{T}\geq 0}{\sup}\phi (\bar{T},x(\bar{T},u)). \end{array}$$

Since *S* ⊂ [0, *T*] × R^*n*^, we know that *T*_*k*_ ≤ *T* for all *k*. Each function *x*_*k*_ can then be extended to the entire interval [0, *T*] by setting *x*_*k*_(*t*) = *x*_*k*_(*T*_*k*_) for *t* ∈ [*T*_*k*_, *T*].

The sequence *x*_*k*_ is uniformly Lipschitz continuous, as *f* is uniformly bounded on bounded sets. This then implies equicontinuity of ${\left\{x_{k}\right\}}_{k=1}^{\infty }$. By the Arzela-Ascoli Theorem, there exists a subsequence *x*_*n*_*k*__ such that *T*_*n*_*k*__ → T_*_, T_*_ ≤ *T*, and *x*_*n*_*k*__ → x_*_ uniformly on [0, T_*_].

Lemma 21 implies that x_*_ is admissible, so that there exists a control u_*_:[0, T_*_] ↦ *U* such that

(121) $$\begin{array}{l} {\dot{x}}_{*}\left(t\right)=f\left(t,x_{*}\left(t\right),u_{*}\left(t\right)\right) \end{array}$$

for almost all *t* ∈ [0, T_*_]. Equations (120) imply that

(122) $$\begin{array}{l} \begin{array}{l} \;x_{*}\left(0\right)=x_{0}\\ \left(T_{*},x_{*}\left(T_{*}\right)\right)=\underset{n_{k}\to \infty }{\lim}\phi \left(T_{n_{k}},x_{n_{k}}\left(T_{n_{k}}\right)\right)\in S. \end{array} \end{array}$$

Note that the second of (122) relies on *S* being closed. Continuity of ϕ and (120) implies that

(123) $$\begin{array}{l} \phi \left(T_{*},x_{*}\left(T_{*}\right)\right)=\underset{n_{k}\to \infty }{\lim}\phi \left(T_{n_{k}},x_{n_{k}}\left(T_{n_{k}}\right)\right)=\underset{u\in U,T_{*}\geq 0}{\sup}\phi \left(T_{*},x\left(T_{*},u\right)\right). \end{array}$$

Thus, u_*_ is optimal, as desired.     □

For the model of drug-induced resistance, the control set *U* is the compact set *U* = [0, *M*], and for such control-affine systems, convexity of *F*(*x*) is implied by the convexity of *U*. Existence of a trajectory satisfying the constraints is clear; for example, take *u*(*t*) ≡ 0. Our objective is to maximize the time to not escape the set *N*. Note that *N* is a closed subset of R^2^, and that

(124) $$\begin{array}{l} \phi \left(\bar{T},x\left(\bar{T},u\right)\right)=\bar{T}. \end{array}$$

is continuous. Lastly, we have seen that all solutions remain in the closure $\bar{\Omega }$, so that |*x*(*t*)| ≤ 1 for all u∈U and hence solutions are uniformly bounded. Existence is then reduced to Item 6 in the previous theorem. Since the supremum of time *t* was shown to be finite, Theorem 22 together with Theorem 19 imply that the optimal control for the problem presented in section 3 exists.

### 7.3. Further Numerical Experiments

In this subsection, we present further numerical experiments (see section 6). Specifically, we study how the values of the relative resistant growth rate and critical volume influence the control structure. We also consider a regularized objective, which suggests that our numerical methods are converging to (at least local) solutions of the optimal control problem.

**Figure 13:**
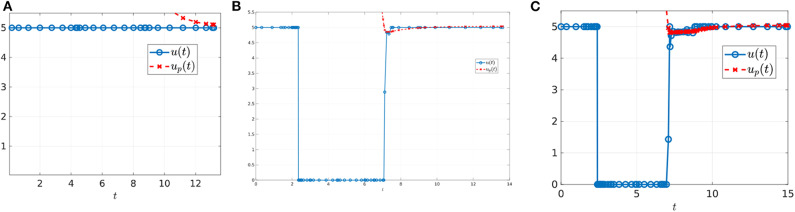
Computed optimal controls for α = 0.005 and **(A)** *d* = 0.0206, **(B)** *d* = 0.020624489795918, and **(C)** *d* = 0.207959. Note that the control in **(A)** takes the form *Y*, while that in **(B,C)** is of the form *YXu*_*p*_.

**Figure 14:**
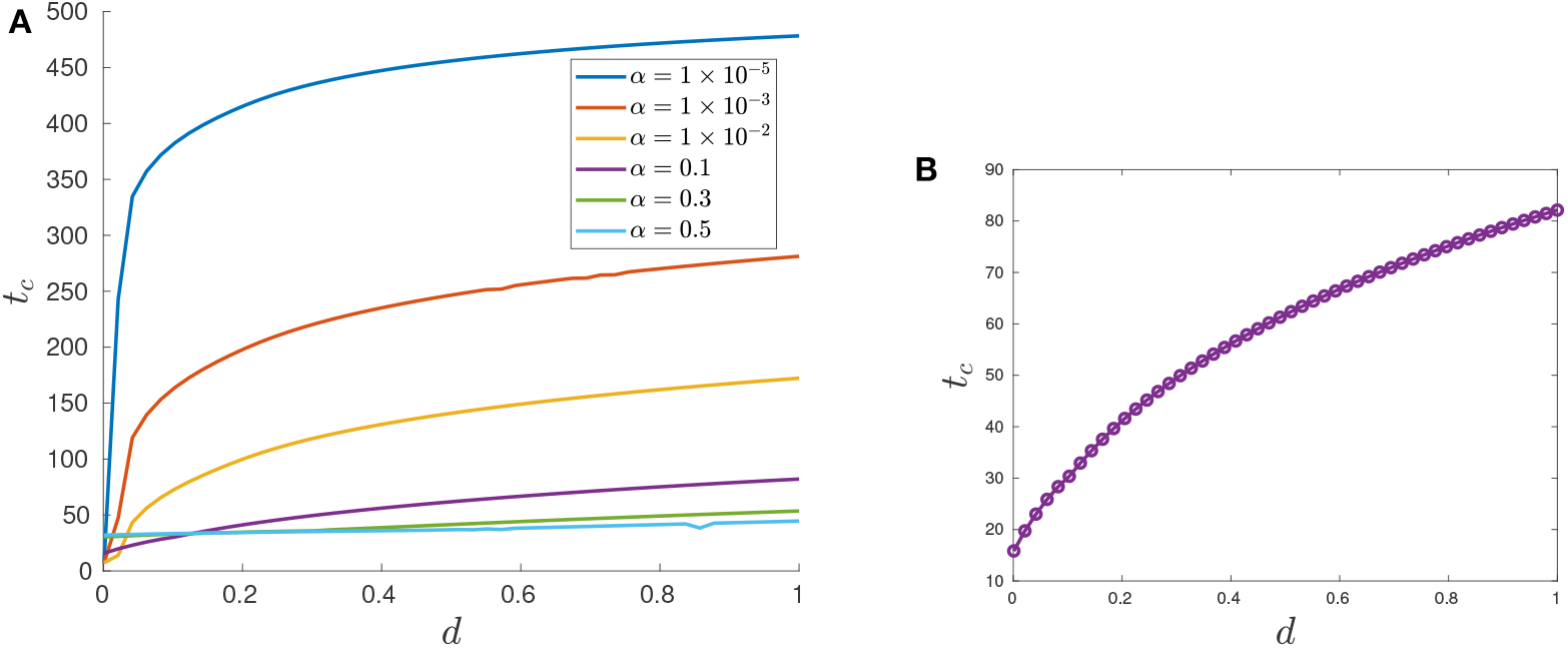
Variation in *t*_*c*_ as a function of *d*. **(A)** *t*_*c*_ response for varying *d* values. Note that treatment efficacy generally increases with increasing *d*. **(B)** α = 0.1.

We first investigate the control structure and treatment outcome as a function of *d* for a fixed α; these results are presented in Figures 13, 14. Here α = 0.005 is fixed and *d* is varied on the interval [0.001, 0.1]. Figure 13 presents three of these controls; although none of the controls is of the form *YXY*, the figure suggests that there may exist a d^*^ ∈ (0.02062, 0.0207959) where the solution trajectory may intersect the boundary line *N* only at one point and subsequently switches into a *Y* arc, thus providing the existence of a *YXY* control. Figure 14 suggests that increasing *d* for a fixed α increases the overall effectiveness of the treatment for all values of α, and that decreasing the induction rate α allows for longer tumor control. However, for small values of *d*, increasing α may provide a better treatment outcome (see, for example, the intersection of the yellow and purple curves in Figure 14).

**Figure 15:**
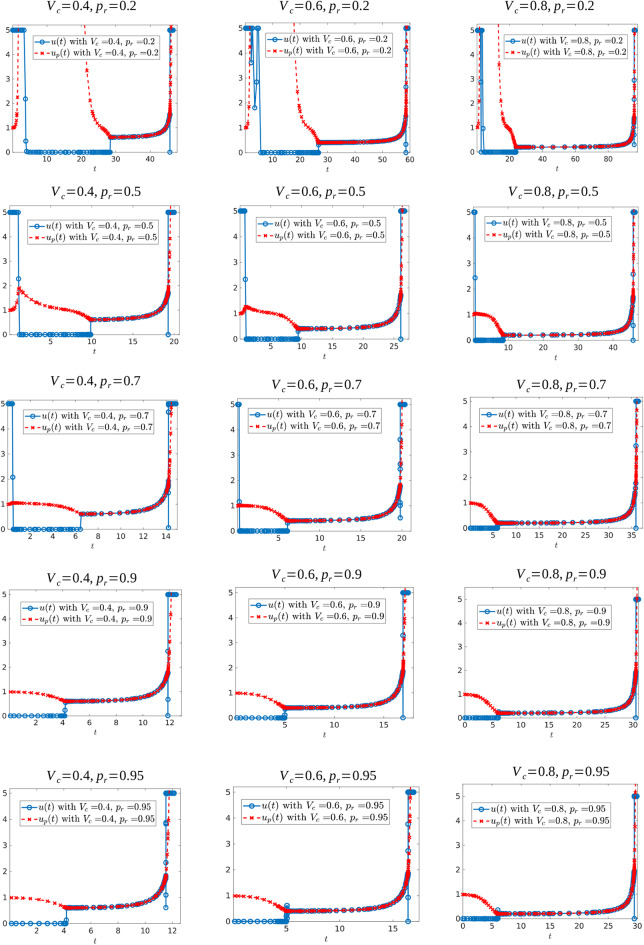
Optimal control structures for different *V*_*c*_ and *p*_*r*_ values. The blue curve is the computed optimal control, while the red curve is the feedback control along on the boundary of *N*, which may or may not be optimal or even feasible.

We also investigated how the shape of the optimal control changes for different values of the resistant growth fraction (*p*_*r*_) and/or the critical tumor volume (*V*_*c*_). We run several simulations for *V*_*c*_ ∈ {0.2, 0.25, 0.3, 0.4, 0.5, 0.6, 0.7, 0.8, 0.85, 0.9} and *p*_*r*_ ∈ {0.2, 0.3, 0.5, 0.7, 0.85, 0.9, 0.95, 0.98, 0.99}. We found that when the reproduction rate of resistant cells is close to the reproduction rate of sensitive cells (*p*_*r*_ near 1), the best strategy is to not give any drug at the beginning of treatment. This is perhaps to prolong the appearance of fast-growing resistance cells which cannot be eliminated with treatment. A representative set of the controls for these simulations are shown in Figure 15.

**Figure 16:**
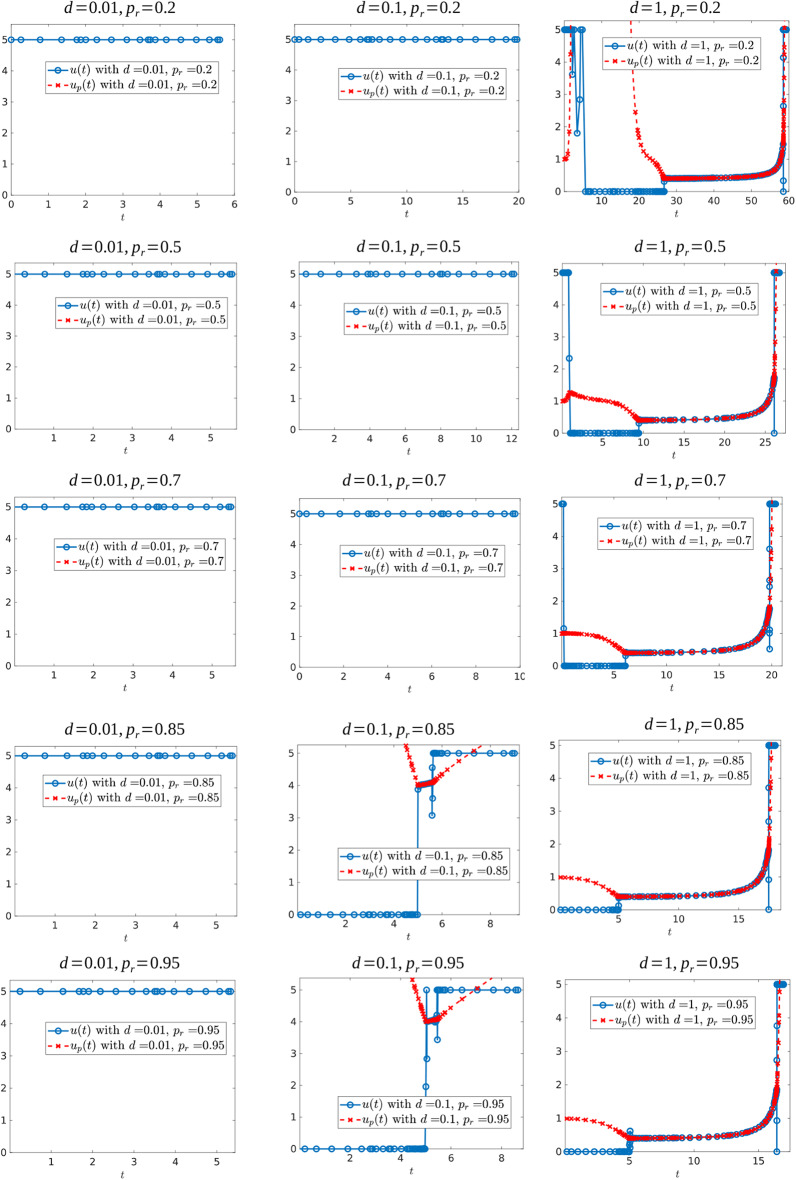
Optimal control structures for *V*_*c*_ = 0.6, and different *d* and *p*_*r*_ values. The blue curve is the computed optimal control, while the red curve is the feedback control along on the boundary of *N*, which may or may not be optimal or even feasible.

**Figure 17:**
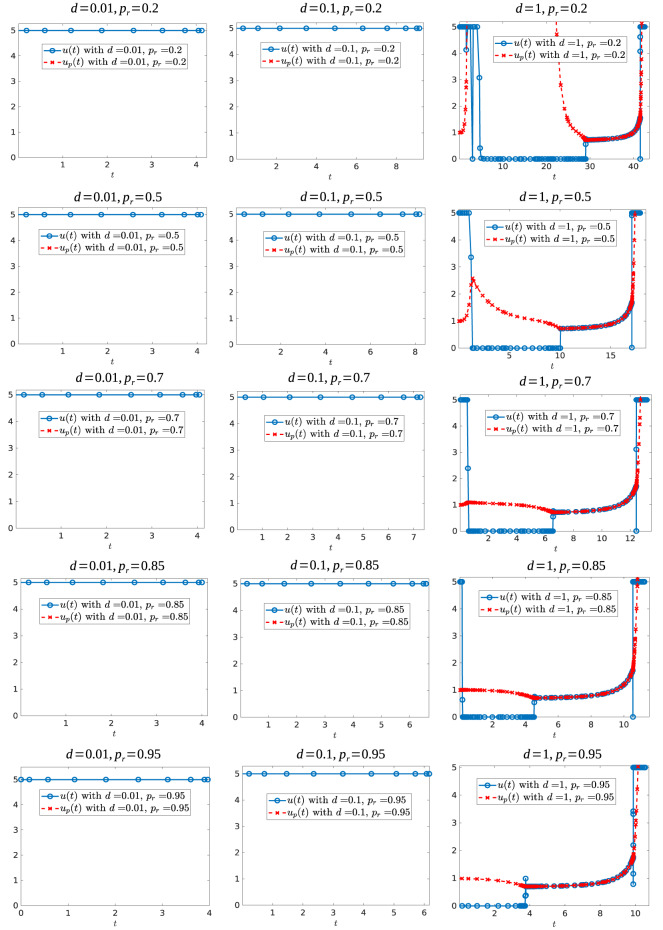
Optimal control structures for *V*_*c*_ = 0.3, and different *d* and *p*_*r*_ values. The blue curve is the computed optimal control, while the red curve is the feedback control along on the boundary of *N*, which may or may not be optimal or even feasible.

We further simulated the following parameter sets: *V*_*c*_ ∈ {0.3, 0.6}, *d* ∈ {0.01, 0.05, 0.1, 0.5, 0.75, 1} and *p*_*r*_ ∈ {0.2, 0.3, 0.5, 0.7, 0.85, 0.9, 0.95, 0.98, 0.99}. Figure 16 shows some of the controls for these simulations for the case when *V*_*c*_ = 0.6, while Figure 17 shows some of the controls for the case *V*_*c*_ = 0.3. In both figures, we observe that independently of the value of resistant growth rate *p*_*r*_, if the chemotherapeutic drug has a low effectiveness (*d* small) then the best strategy is to give the maximum possible drug dosage during treatment. However, when *d* increases past *d* = 0.1, the control structure changes qualitatively. When *V*_*c*_ = 0.6 and the resistant reproduction rate is close to the reproduction rate of sensitive cells, the best strategy is to start with no drug treatment while for case *V*_*c*_ = 0.3 (independently of the value of *p*_*r*_) the best strategy is to give the maximum drug dosage from the start.

Before ending this section, we would like to mention that to verify the performance of the numerical software, we approached the original problem by a sequence of regularized problems, which is done by adding a quadratic term to the Lagrangian. More precisely, we considered the perturbed performance index:

(125) $$J_{\eta }[u]=-\int_{0}^{t_{c}}\left[1-\frac{\left(1-\eta \right)}{2}u^{2}(t)\right]dt\text{ for }\eta \in [\text{0},\text{1}].$$

**Figure 18:**
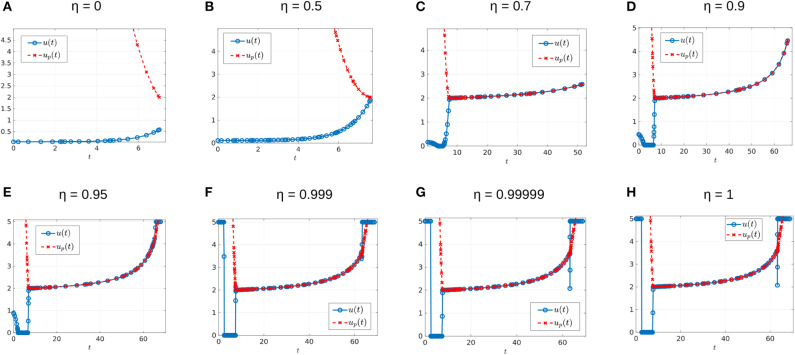
Different perturbed controls for α = 0.005 and *d* = 0.05. Here, from **(A–H)**, the value of η is 0, 0.5, 0.7, 0.9, 0.95, 0.999, 0.99999, and 1, respectively. The maximum relative error is of 4.0338 × 10^−7^ for figure η = 0, the remaining figures have a maximum relative error of 5.5727 × 10^−7^ or smaller.

Notice that Equation (125) represents a family of performance indexes parameterized by η. The original performance index corresponds to η = 1. Furthermore, for η ≠ 1 the optimal control problem is regular and solvers such as GPOPS-II (used here) or SNOPT should provide accurate solutions. Thus, to test the accuracy to the case η = 1, we investigated the corresponding control structure in the limit η → 1. An example of different controls, for η values 0, 0.5, 0.7, 0.9, 0.95, 0.999, 0.99999, and 1, are shown on Figure 18. For each case we obtained different relative errors: the largest relative error of 4.0338 × 10^−7^ occurs for η = 0, with the remaining values of η having smaller relative errors. From the values η = 0.95, η = 0.999 and η = 0.99999 in Figure 18 we can see that as η → 1 the computed control approaches the solution to the original problem (case η = 1).

## 8. Conclusions

In this work, we have provided a rigorous analysis of the optimal control problem introduced in Greene et al. (2018a). That is, we have formally applied optimal control theory techniques to understand treatment strategies related to a model of induced drug resistance in cancer chemotherapy introduced in Greene et al. (2019). Although the model is relatively simple, it has recently been found to be highly successful in matching experimental data (Gevertz et al., 2019; Johnson et al., 2020), which we believe justifies the careful analysis presented here. An optimal control problem is then presented which maximizes a specific treatments therapy window. A formal analysis of the optimal control structure is performed utilizing the Pontryagin Maximum Principle and differential-geometric techniques. Optimal treatment strategies are realized as a combination of bang-bang and path-constrained arcs, and singular controls are proved to be sub-optimal. Numerical results are presented which verify our theoretical results, and demonstrate interesting and non-intuitive treatment strategies. We have also shown that a drug's level of resistance induction is identifiable, thus allowing for the possibility of designing therapies based on individual patient-drug interactions (see section 7.1).

Under the assumption that sensitive cells have a higher growth rate than resistant cells, our results (section 6) indicate that when using a chemotherapeutic drug with low cytotoxicity, the time at which the tumor volume exceeds its critical value *t*_*c*_ would be larger when the transition rate of the drug is high (see for example Table 2, on cases *d* = 0.001 and *d* = 0.01, as α has larger values the end time *t*_*c*_ becomes larger). The situation is reversed when we consider larger values of drug effectiveness because in this case it would take more time for the tumor to grow to its critical volume whenever the drug effectiveness is large enough. Also, our simulations indicate that it is optimal to apply the maximal dosage *M* subsequent to sliding along the boundary *V* = *V*_*c*_ (e.g., Figure 9), prior to treatment failure.

Clearly, further analysis is required in order to understand this phenomenon, and its implications for clinical scenarios. Although our model considers only an idealized scenario where resistance is unavoidable, we see that induced resistance dramatically alters therapy outcome, which underscores the importance of understanding its role in both cancer dynamics and designing chemotherapy regimes.

**Figure 19:**
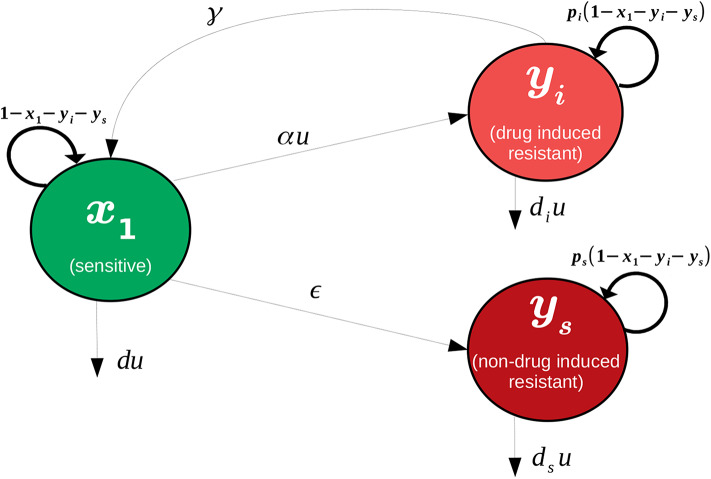
Visualization of model that includes a reverse phenotype transition from resistant to sensitive. *x*_1_ denotes the sensitive cancerous cell population, *y*_*i*_ the drug-induced resistant cancerous cell population, and *y*_*s*_ the non-drug-induced resistant cell population.

**Figure 20:**
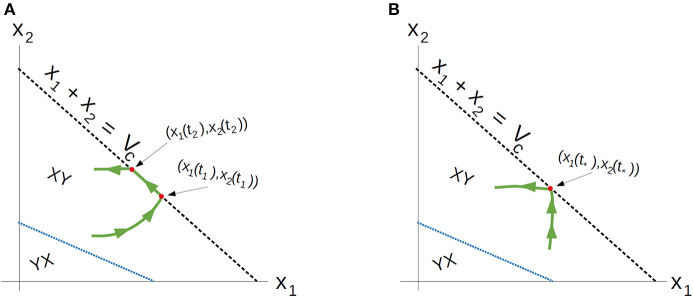
**(A)** Example of an arc with feedback control with entry point [*x*_1_(*t*_1_), *x*_2_(*t*_1_)] an exit point [*x*_1_(*t*_2_), *x*_2_(*t*_2_)] the exit point **(B)** Example of an arc that does not slides but reaches the boundary *V* = *V*_*c*_ at the contact point (*x*_1_(t_*_), *x*_2_(t_*_)).

Other questions remain open for future work:

♢ Several studies indicate that drug-tolerance is a phenotypic property that appears transiently under the presence of the drug (Goldman et al., 2015). A next step to this research is to incorporate a reverse transition rate (from resistant to sensitive cells) that represents this phenotype-switching (see Figure 19).
♢ For controls where the trajectory remains on the boundary *V* = *V*_*c*_ (*u*_*p*_), the feedback control is optimal during a time interval [*t*_1_, *t*_2_] with 0 ≤ *t*_1_ < *t*_2_ < *t*_*c*_. It remains to understand the point of entry [*x*_1_(*t*_1_), *x*_2_(*t*_1_)] and exit [*x*_1_(*t*_2_), *x*_2_(*t*_2_)] (Figure 20A). What is the significance of the times *t*_1_ and *t*_2_ with respect to parameter values?
♢ Do there exist conditions, once the trajectory reaches *V*_*c*_, under which the optimal trajectory no longer slides? Is it possible that at the time t_*_ the point [*x*_1_(t_*_), *x*_2_(t_*_)] is a contact point (Figure 20B)? Some numerical results suggest that such a contact point may exist and give rise to a *YXY* control structure (Figure 13).
♢ We have shown that an optimal control can switch at most once in each of the regions $\Omega_{c}^{+}$ and $\Omega_{c}^{-}$. Numerically we did not observe any bang-bang controls of the form *YXY*, although its existence was strongly suggested. The existence of a bang-bang junction in $\Omega_{c}^{-}$ is therefore of interest.
♢ For all examples plotted in Figure 11 with *d* ≥ 0.1, the entry time occurs approximately at the same value *t*_1_ = 20.03. Is this a coincidence? We would like to understand the dependence of the entry time *t*_1_ and on parameters α, *d*, *p*_*r*_, *M*, and/or ϵ.
♢ We would like to extend models to include multiple, possibly non-cross resistant, cytotoxic agents. Indeed, clinical practice generally includes multiple agents applied concurrently and sequentially, and we plan on investigating strategies when different types of drugs may be applied. For example, what control strategies arise when a targeted therapy exists which targets the resistant sub-population? What order should the agents be applied, and for how long? Are intermediate doses now optimal? Mathematically, all of these questions may be studied, and the results may be clinically relevant.

## Author Contributions

All authors listed have made a substantial, direct and intellectual contribution to the work, and approved it for publication.

## Conflict of Interest

The authors declare that the research was conducted in the absence of any commercial or financial relationships that could be construed as a potential conflict of interest.
